# Adaptation process of decellularized vascular grafts as hemodialysis access *in vivo*

**DOI:** 10.1093/rb/rbae029

**Published:** 2024-03-21

**Authors:** Tun Wang, Peng Lu, Zicheng Wan, Zhenyu He, Siyuan Cheng, Yang Zhou, Sheng Liao, Mo Wang, Tianjian Wang, Chang Shu

**Affiliations:** Department of Vascular Surgery, The Second Xiangya Hospital, Central South University, Changsha 410011, China; Institute of Vascular Diseases, Central South University, Changsha 410011, China; Department of Vascular Surgery, The Second Xiangya Hospital, Central South University, Changsha 410011, China; Institute of Vascular Diseases, Central South University, Changsha 410011, China; Department of Vascular Surgery, The Second Xiangya Hospital, Central South University, Changsha 410011, China; Institute of Vascular Diseases, Central South University, Changsha 410011, China; Department of Vascular Surgery, The Second Xiangya Hospital, Central South University, Changsha 410011, China; Institute of Vascular Diseases, Central South University, Changsha 410011, China; Department of Vascular Surgery, The Second Xiangya Hospital, Central South University, Changsha 410011, China; Institute of Vascular Diseases, Central South University, Changsha 410011, China; Department of Vascular Surgery, The Second Xiangya Hospital, Central South University, Changsha 410011, China; Institute of Vascular Diseases, Central South University, Changsha 410011, China; Department of Vascular Surgery, The Second Xiangya Hospital, Central South University, Changsha 410011, China; Institute of Vascular Diseases, Central South University, Changsha 410011, China; Department of Vascular Surgery, The Second Xiangya Hospital, Central South University, Changsha 410011, China; Institute of Vascular Diseases, Central South University, Changsha 410011, China; Department of Vascular Surgery, The Second Xiangya Hospital, Central South University, Changsha 410011, China; Institute of Vascular Diseases, Central South University, Changsha 410011, China; Department of Vascular Surgery, The Second Xiangya Hospital, Central South University, Changsha 410011, China; Institute of Vascular Diseases, Central South University, Changsha 410011, China; Center of Vascular Surgery, Fuwai Hospital, Chinese Academy of Medical Sciences and Peking Union Medical College, Beijing 100037, China

**Keywords:** arteriovenous graft, decellularized vascular grafts, animal model, *in vivo* revascularization, immune cell infiltration

## Abstract

Arteriovenous grafts (AVGs) have emerged as the preferred option for constructing hemodialysis access in numerous patients. Clinical trials have demonstrated that decellularized vascular graft exhibits superior patency and excellent biocompatibility compared to polymer materials; however, it still faces challenges such as intimal hyperplasia and luminal dilation. The absence of suitable animal models hinders our ability to describe and explain the pathological phenomena above and *in vivo* adaptation process of decellularized vascular graft at the molecular level. In this study, we first collected clinical samples from patients who underwent the construction of dialysis access using allogeneic decellularized vascular graft, and evaluated their histological features and immune cell infiltration status 5 years post-transplantation. Prior to the surgery, we assessed the patency and intimal hyperplasia of the decellularized vascular graft using non-invasive ultrasound. Subsequently, in order to investigate the *in vivo* adaptation of decellularized vascular grafts in an animal model, we attempted to construct an AVG model using decellularized vascular grafts in a small animal model. We employed a physical–chemical–biological approach to decellularize the rat carotid artery, and histological evaluation demonstrated the successful removal of cellular and antigenic components while preserving extracellular matrix constituents such as elastic fibers and collagen fibers. Based on these results, we designed and constructed the first allogeneic decellularized rat carotid artery AVG model, which exhibited excellent patency and closely resembled clinical characteristics. Using this animal model, we provided a preliminary description of the histological features and partial immune cell infiltration in decellularized vascular grafts at various time points, including Day 7, Day 21, Day 42, and up to one-year post-implantation. These findings establish a foundation for further investigation into the *in vivo* adaptation process of decellularized vascular grafts in small animal model.

## Graphical abstract

**Figure:**
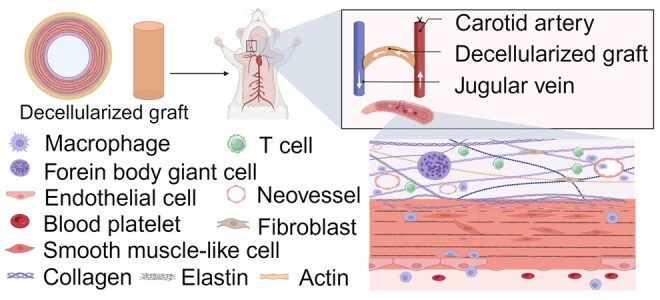

## Introduction

Chronic kidney disease (CKD) is an increasingly recognized global public health issue, with a 40% increase in incidence over the past 30 years [1]. Hemodialysis access is the ‘lifeline’ for patients with end-stage CKD, and arteriovenous grafts (AVGs) are the preferred choice for constructing hemodialysis access in many patients [2]. However, multiple research studies have shown that the current commonly used expanded polytetrafluoroethylene (ePTFE) artificial grafts have a primary patency rate of less than 50%, resulting in a significant economic burden on patients [2, 3]. The ePTFE artificial graft is essentially a chemical material polymer and lacks the capability for biological regulation within the body. Consequently, its interior cannot support cell regeneration, preventing the process of neovascularization [4]. We need to develop superior materials for vascular grafts.

Decellularized vascular grafts refer to natural blood vessels or other tissues that have been stripped of cellular components (reducing antigenicity) using physical, chemical, or biological methods, while retaining extracellular matrix components such as collagen and elastin to provide sufficient mechanical properties [5–7]. Both decellularized vascular grafts and acellular human vascular grafts primarily consist of extracellular matrix components, resulting in significantly better tissue compatibility and superior revascularization capabilities compared to ePTFE artificial grafts [8, 9]. Clinical trials on decellularized vascular grafts have demonstrated comparable primary patency and primary assisted patency rates at 1 and 2 years and higher secondary patency rate when compared to ePTFE artificial grafts [10, 11]. The results of the Phase II clinical trial of human acellular vascular grafts (HAVs) developed by Lawson *et al*. [12] showed a primary patency rate of 28% within 1 year and a primary assisted patency rate of 38%.

Current research findings indicate that both decellularized vascular grafts and HAVs are facing challenges such as rejection reactions, intimal hyperplasia, suboptimal revascularization and other issues [8, 13]. Limited evidence suggests the involvement of immune cells, including macrophages, in the graft adaptation process *in vivo* [14]. Decellularized vascular grafts possess unique tissue structures [15], and it is still unknown whether the molecular mechanisms underlying intimal hyperplasia (or other phenomena) after their transplantation are consistent with those resulting from other pathological conditions such as arteriovenous fistulas (AVF), intimal injuries, and atherosclerosis. Due to the lack of suitable small animal models, current research has primarily focused on *in vitro* material characterization and *in vivo* experiments using large animals. Consequently, our understanding of the mechanisms underlying the *in vivo* adaptation process of decellularized vascular graft materials remains limited. Currently, there is no systematic description available regarding the process of decellularized vascular graft implantation *in vivo*. Further investigation into the mechanisms underlying various phenomena observed after the *in vivo* transplantation of decellularized vascular grafts would greatly enhance the effectiveness of this technique.

We have innovatively established the allogeneic rat carotid arteriovenous graft model using decellularized vascular grafts. This model demonstrates excellent patency rates, high success rates, and exhibits phenotypic similarities to clinical scenarios. Using this model, we have described the *in vivo* adaptation process of using decellularized vascular grafts for the first time. For details, refer to the research schematic.

## Materials and methods

### Decellularization of native iliac artery

Decellularization of native iliac artery was accomplished as described previously [16]. Briefly, iliac artery was incubated in 22 h in 3-[(3-Chloamidopropyl)dimethyl- ammonium]-1- propanesulfonate (CHAPS) buffer (8 mM CHAPS [Sigma–Aldrich, St Louis, MO], 1 M NaCl [Sigma–Aldrich], and 25 mM Ethylenediaminetetraacetic acid (EDTA) [Sigma–Aldrich] in PBS), followed by 22 h in Sodium dodecyl sulfate (SDS) buffer containing 1.8 mM SDS (Sigma–Aldrich), 1 M NaCl and 25 mM EDTA in PBS, 48 h of phosphate buffered solution (PBS) washes. We further subjected the decellularized vascular grafts to DNase I (3000 U/ml) and RNase A (3000 U/ml) treatment for 1 h. Decellularized iliac artery was stored in PBS containing penicillin (100 U/ml) and streptomycin (0.1 mg/ml) and placed at 4°C until use.

### Decellularization of rat carotid artery

The right rat carotid artery (CA) (1.5 cm length) from male Sprague-Dawley (SD) rats (9 week) was dissected and carefully removed using sterile technique; the CA were then stored at 4°C in PBS containing penicillin 100 U/ml and streptomycin 100 mg/ml (Invitrogen). Decellularization of CA was accomplished as described previously [17]. Physical method: Refers to cryopreservation of vascular by placing the CA in a −20°C refrigerator overnight. Chemical method: Involves decellularization of the CA using CHAPS and sodium dodecyl sulfate. Briefly, CA was incubated in CHAPS buffer (8 mM CHAPS, 1 M NaCl and 25 mM EDTA in PBS) for 12 h, followed by a 60-min wash. Subsequently, it was incubated in sodium dodecyl sulfate buffer (1.8 mM sodium dodecyl sulfate, 1 M NaCl and 25 mM EDTA in PBS) for 24 h, followed by a 24-h wash with PBS to completely remove the detergent. Biological method: Involves further removal of potential nucleic acid macromolecules using DNase and RNase. Specifically, CA was incubated in PBS containing DNase I (3000 U/ml) and RNase A (3000 U/ml) at room temperature for 1 h, followed by a 60-min wash. The ‘Physical–Chemical–Biological Protocol’ refers to a combined approach incorporating the three aforementioned methods and represents the ultimate decellularization strategy employed in this study. The decellularized CA were then stored at 4°C in PBS containing penicillin 100 U/ml and streptomycin 100 mg/ml.

### Scanning electron microscope

The samples were fixed in 2.5% (v/v) glutaraldehyde for 12 h and dehydrated in ascending series of ethanol. Then, the samples were mounted onto aluminum stubs, sputter-coated with gold, and observed by SEM (Phenom Pro, Phenom-World BV, Eindhoven, the Netherlands).

### Animal models

#### Animals

All experiments were approved by the Animal Care and Use Committee of the Second Xiangya Hospital of Central South University (Approval No. 20231043). All experiments were performed in accordance with the National Institutes of Health Guide for the Care and Use of Laboratory Animals. Male SD rats at 9 weeks of age were selected for modeling. Younger SD rats have smaller diameters of the carotid artery and jugular vein, which increase the surgical difficulty and are unable to tolerate prolonged surgeries.

#### Construction of the renal failure model

The SD rats were fed with feed containing 0.25% adenine for approximately 21 days. Throughout the feeding period, the rats’ health status was regularly observed, including monitoring changes in body weight, food intake and urine volume. Blood samples were collected for the determination of biochemical markers to assess the extent of renal impairment. Additionally, histopathological examination of renal tissues was performed to confirm the successful establishment of the model.

#### Surgical procedure

The rat carotid artery–jugular vein AVG model requires technical skills and microvascular surgical expertise. We recommend having at least one trained surgeon perform the surgical procedures. Detailed surgical procedure can be found in the Supplementary materials.

#### Follow-up using non-invasive Doppler ultrasound

We used non-invasive Doppler ultrasound to monitor the vascular patency, hemodynamic changes and lumen diameter in our model at Days 7, 21, 42 and 1 year after surgery. Technically, weekly Doppler ultrasound can be performed and is recommended to monitor changes in hemodynamics until the animal is sacrificed.

### Histology

The acquisition of all tissues was approved by the Medical Ethics Committee of the Second Xiangya Hospital (Approval No. 2019-Clinical Research-82), and patients provided informed consent for the use of the samples. Samples was immediately rinsed in PBS and then immersed in 4% paraformaldehyde for fixation as soon as the tissue was removed. The tissue was then dehydrated in ethanol and xylene and then embedded in paraffin. The tissue was cut in 4-μm cross sections. Hematoxylin eosin (HE) staining was used to assess histomorphology (HE kits, Servicebio, G1005). For Extracellular Matrix (ECM) remodeling and quantification, tissue sections were stained with Masson’s Trichrome to measure collagen density (Solarbio Life Sciences, G1340). Elastin Van Gieson (EVG) staining was used to measure intima-media thickness and to detect the integrity of elastic fibers. (Hao Ke Biotechnology Co. Ltd, HKT2034).

### Immunohistochemistry

Tissue sections were de-paraffined using xylene and a graded series of alcohols. Sections were heated in 0.01 M citric acid buffer (pH 6.0) at 100°C for 20 min and cooled to room temperature. After washing three times by PBS, sections were blocked with 5% normal goat serum in PBS (pH 7.4) for 1 h at room temperature. Sections were then incubated at 4°C overnight with the primary antibody anti-α-SMA antibody (Abcam, ab7817,1:100), anti-CD68 antibody (Abcam, ab955,1:100) and anti-CD3 antibody (Abcam, ab16669,1:100). Secondary antibodies were goat anti-rabbit a conjugated antibody (abcam). Sections were stained with 3,3-N-Diaminobenzidine tetrahydrochloride.

### Counting of infiltrating cells within elastic fibers

Initially, EVG staining was employed to label elastic fibers, followed by the use of hematoxylin and eosin (H&E) staining with hematoxylin marking cell nuclei for cellular localization. Subsequently, the number of cell nuclei within the elastic fibers were identified and quantified to represent the extent of cellular infiltration. To ensure data accuracy, immunofluorescence staining was also performed for counting. Elastic fibers were visualized under overexposure to green fluorescence, and 4′,6-diamidino-2-phenylindole (DAPI) was used to stain cell nuclei. Subsequently, the number of cell nuclei within the elastic fibers were identified and quantified to represent the extent of cellular infiltration.

### Immunofluorescence

Tissue sections were de-paraffined and then heated in citric acid buffer (pH 6.0) at 100°C for 15 min for antigen retrieval. The sections were blocked with 5% bovine serum albumin for 60 min at room temperature, and then incubated with primary antibody overnight at 4°C. For clinical samples, anti-CD31 antibody (Abcam, ab9498,1:100), anti-CD68 antibody (Abcam, ab955,1:100) were used. For rat samples, anti-CD31 antibody (Abcam, ab222783, 1:100), anti-Proliferating Cell Nuclear Antigen (PCNA) antibody (Cell signaling, 13110 T, 1:100), anti-CD68 antibody (Abcam, ab31630, 1:100) were used. For both clinical and rat samples, anti-α-SMA antibody (Abcam, ab7817, 1:100), anti-CD3 antibody (Abcam, ab16669, 1:100) were used. After incubation, the sections were incubated with Alexa Fluoro secondary antibodies for 1 h and stained with DAPI (P36935, Invitrogen) to stain cellular nuclei. Positively staining cells were counted per high power fields or measured the intensity. For negative controls for the antibodies, IgG isotype controls, negative tissue controls and endogenous tissue background controls were used.

### TUNEL assay

Cell apoptosis in tissues was measured using TUNEL assay (Abcam, ab66110). Apoptotic cells of each group were counted from five random fields of view.

### Statistics

Data are represented as mean value ± standard error of the mean. All data were analyzed using Prism 8 software (GraphPad Software, Inc., La Jolla, CA). Equal variance was confirmed using the Shapiro–Wilk test prior to performing parametric analyses. Statistical significance was determined using Student’s *t*-test or ANOVA with Sidak’s *post hoc* correction. We used the Mann–Whitney U test or the Kruskal–Wallis test with Dunn’s *post hoc* correction if the sample size was smaller than 6. *P* values < 0.05 were considered significant. * *P* ≤ 0.05, ** *P* ≤ 0.01, *** *P* ≤ 0.001.

## Results

### Evaluation of decellularized graft 5 years post-implantation in human

**Figure 1.**
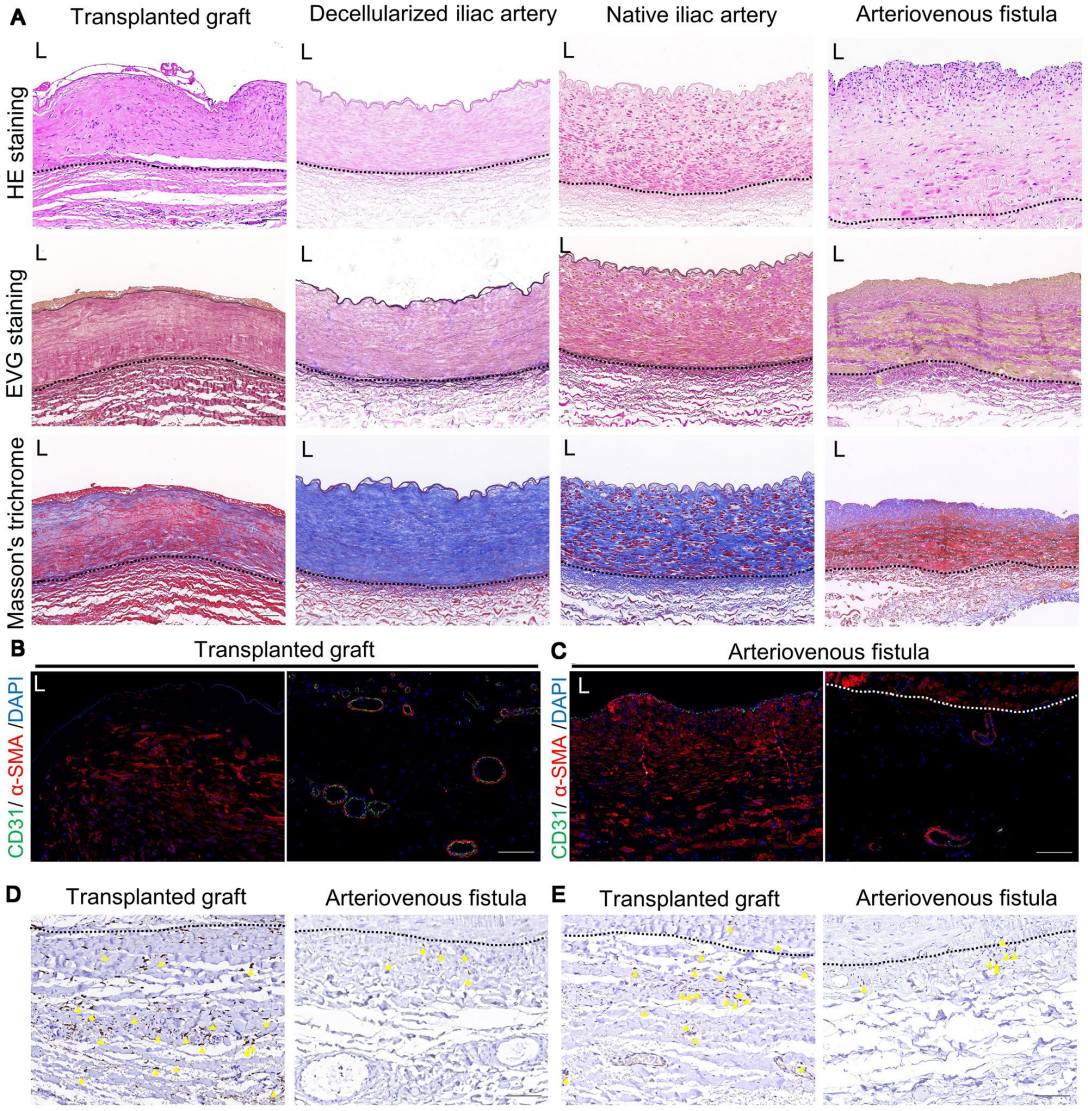
Evaluation of allogeneic decellularized graft in the human body 5 years post-implantation. (**A**) Hematoxylin and eosin (HE), Verhoeff’s Van Gieson (EVG) staining and Masson’s trichrome staining of transplanted graft, decellularized iliac artery, native iliac artery and arteriovenous fistula (AVF) (scale bar, 100 μm). (**B**) Immunofluorescence was used to assess the expression of CD31 and α-SMA in the intima-media (left) and adventitia (right) of transplanted graft (scale bar, 100 μm). (**C**) Immunofluorescence was used to assess the expression of CD31 and α-SMA in the intima-media (left) and adventitia (right) of AVF (scale bar, 100 μm). (**D**) Immunohistochemistry (IHC) was used to assess the expression of CD68 in the adventitia of both the transplanted graft and AVF (scale bar, 100 μm). (**E**) IHC was used to assess the expression of CD3 in the adventitia of both the transplanted and AVF (scale bar, 100 μm). L represents the lumen, the area outside the dashed lines represents adventitia area of native vascular or transplanted graft, and the yellow triangle represents cells with positive expression.

We encountered a patient in the late stage of chronic kidney failure who had previously undergone the construction of an arteriovenous fistula using an allogeneic decellularized iliac artery. Unfortunately, the specific details of the arteriovenous fistula surgery are not known. The patient required a fistula closure procedure due to undergoing a kidney transplant. After ethical evaluation and obtaining informed consent from the patient, the decellularized vascular graft which had been previously used in the human body for 5 years, hereafter referred to as ‘transplanted graft,’ was obtained. The native iliac artery specimens were obtained from patients requiring amputation surgery. The decellularized iliac artery was prepared by removing the cellular components from the native iliac artery. The acquisition of all tissues has obtained approval from the Ethics Committee and informed consent from the patients. We conducted histological evaluations of the transplanted graft, decellularized iliac artery, and native iliac artery. In order to investigate whether the *in vivo* situation of the decellularized graft is similar to that of an arteriovenous fistula (AVF), we also performed histological evaluations of the AVF outflow tract (Figure 1A).

The results indicate that after decellularization, the cellular structure within the decellularized iliac artery has disappeared, but the extracellular matrix components are well-preserved. Five years after implantation, the cellularization results of the transplanted graft were quite satisfactory. In the HE staining results, more cell nuclei were observed, while EVG staining showed that there was a slight degradation of the outer elastic fibers of the transplanted graft, but the main elastic fibers were well-preserved. Masson staining revealed that compared to the decellularized iliac artery, the transplanted graft exhibited obvious smooth muscle fiber presence (appearing in red in Masson staining). Furthermore, we did not observe significant intimal hyperplasia in the transplanted graft *in vivo*, although this may be due to the sample's location being in the middle of the graft, away from the two ends of the graft. However, we observed significant intimal hyperplasia in the AVF sample, which is one of the main causes of AVF occlusion. These results indicate that the decellularized iliac artery has undergone a satisfactory adaptation process in the body after 5 years of use.

Furthermore, we assessed the expression of CD31 and α-SMA in both the transplanted graft and AVF outflow tract (Figure 1B and C), as well as the expression of CD68 and CD3 (Figure 1D and E). We observed a significant number of α-SMA-positive cells within the transplanted graft, which appeared to be spindle-shaped arranged. The inner layer of the transplanted graft lacks coverage of CD31 positive cells (Figure 1B), which may be due to the loss of endothelial cells during the sample acquisition caused by flushing and clamping during the surgical procedure. In the outer layer of natural blood vessels, there are small blood vessels called adventitial vasa vasorum, which provide nutrients to the vessel wall and may also serve as pathways for immune cell infiltration under disease conditions [18]. We observed a significant number of neovessels within the outer layer of the transplanted graft (Figure 1B). These neovessels are very similar to the adventitial vasa vasorum in normal blood vessels but are more abundant in quantity, far exceeding the number of adventitial vasa vasorum in the AVF.

In natural blood vessels, a small number of immune cells, such as tissue-resident macrophages, are known to be present [19]. The roles of macrophages and T cells in AVF tissue have been established [20, 21]. In our study, we observed a significant infiltration of macrophages in the transplanted graft, with a higher number of macrophages compared to the AVF (Figure 1D). We also observed T cell infiltration in the inner region of the transplanted graft, near the blood vessel lumen, whereas no T cell infiltration was found in the AVF’s middle and newly formed intimal layers (Figure 1E and Supplementary Figure S2). Consistent with the AVF, the outer layer of the transplanted graft showed higher infiltration of T cells (Figure 1E). This difference in macrophages and T cell infiltration may be attributed to the chronic inflammatory response triggered by the graft implantation. The impact of these immune cells on the adaptation process of decellularized vascular grafts in the body remains unclear. However, these findings suggest that immune regulation might play a role in improving the utility of decellularized vascular grafts.

### The non-invasive ultrasound evaluation of the decellularized vascular graft in human

**Figure 2.**
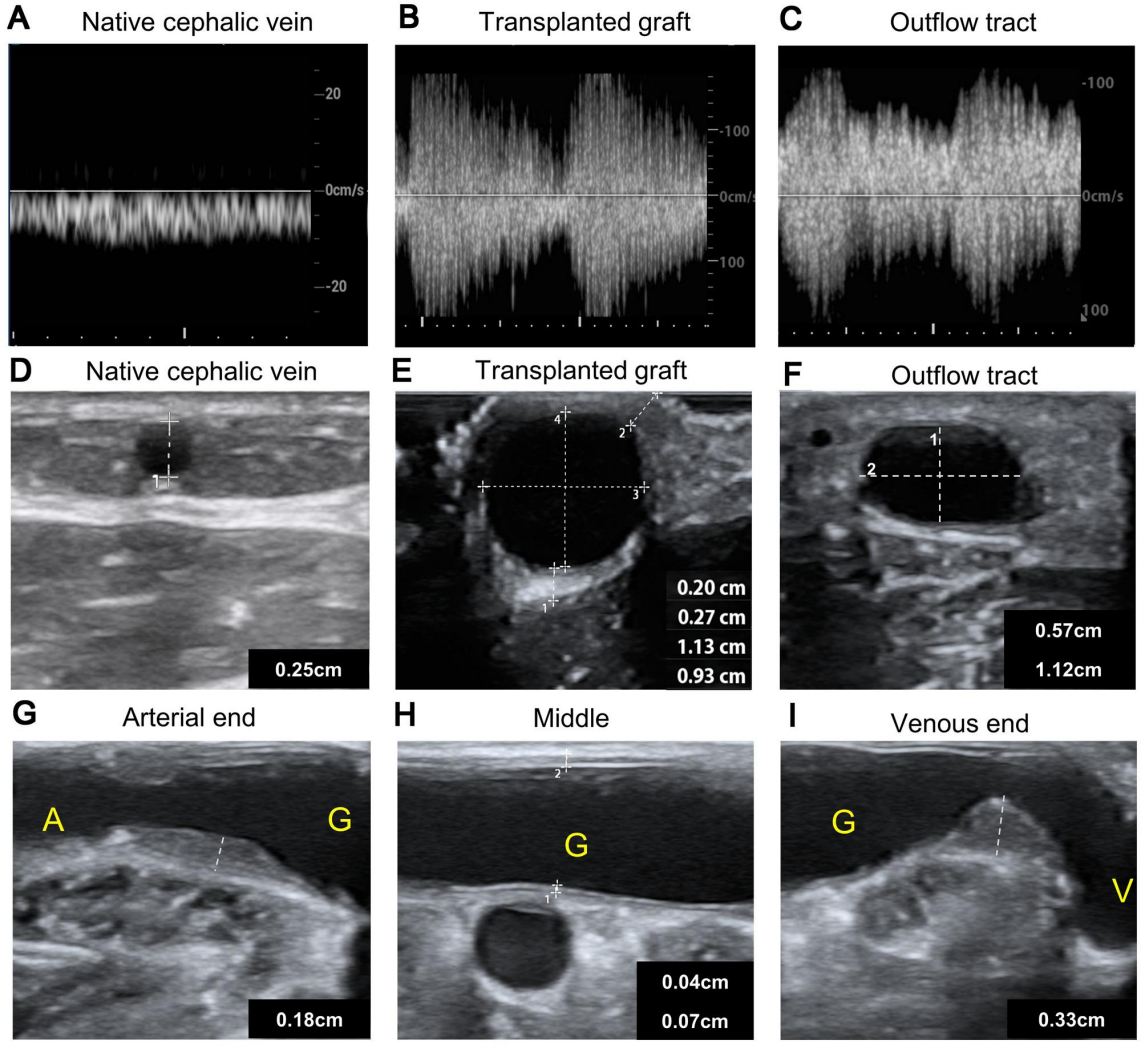
The non-invasive ultrasound evaluation of the decellularized vascular graft in human. (**A–C**) The ultrasound spectra of the cephalic vein (A), decellularized graft (B) and outflow tract of decellularized graft (C). (**D**–**F**) The vascular diameters of the cephalic vein (D), transplanted graft (E) and outflow tract of decellularized graft (F). (**G**–**I**) the extent of intimal hyperplasia at the arterial anastomotic end (G), Middle segment (H) and venous anastomotic end (I) of the decellularized graft. A represents the artery connected to the graft; G represents the graft; ‘V’ represents the vein outflow tract connected to the graft.

Prior to obtaining tissues from patients, we performed non-invasive ultrasound evaluations on the *in vivo* condition of the transplanted grafts (Figure 2 and Supplementary Figure S1). The transplanted graft exhibited significant arterial-like spectra and demonstrated rapid blood flow velocities (Figure 2B). The outflow tract of connected to the transplanted graft also displayed similar arterial-like spectra (Figure 2C). These findings indicate excellent patency of the decellularized grafts. Both the transplanted graft and the autogenous vein had larger diameters compared to the native cephalic vein (Figure 2D and F). Notably, the decellularized iliac artery did not show significant dilation 5 years post-implantation, as opposed to the native iliac artery, which is typically around 1 cm in diameter. This suggests that the decellularized graft possesses favorable mechanical properties, enabling it to withstand high wall shear stresses over an extended period (Figure 2E). Using non-invasive ultrasound, we approximated the thickness of the transplanted graft to be within the range of 0.20–0.27 cm (Figure 2E). Furthermore, the autogenous vein connected to transplanted graft underwent postoperative dilation, meeting the maturity criteria of an arteriovenous fistula (AVF) (Figure 2F) [2].

Intimal hyperplasia-induced luminal narrowing at the anastomotic site is a primary factor leading to AVF and AVG occlusion [4, 22]. In our study, we observed a similar phenomenon of intimal hyperplasia occurring at the anastomotic sites of the transplanted graft *in vivo* (Figure 2G–I). The degree of intimal hyperplasia was more pronounced at the graft ends compared to the middle segment. Notably, the neointima at the venous anastomotic site occupied a considerable portion of the lumen. These observations emphasize the significance of regulating intimal hyperplasia in decellularized vascular grafts.

### The evaluation of rat carotid artery before and after decellularization

**Figure 3.**
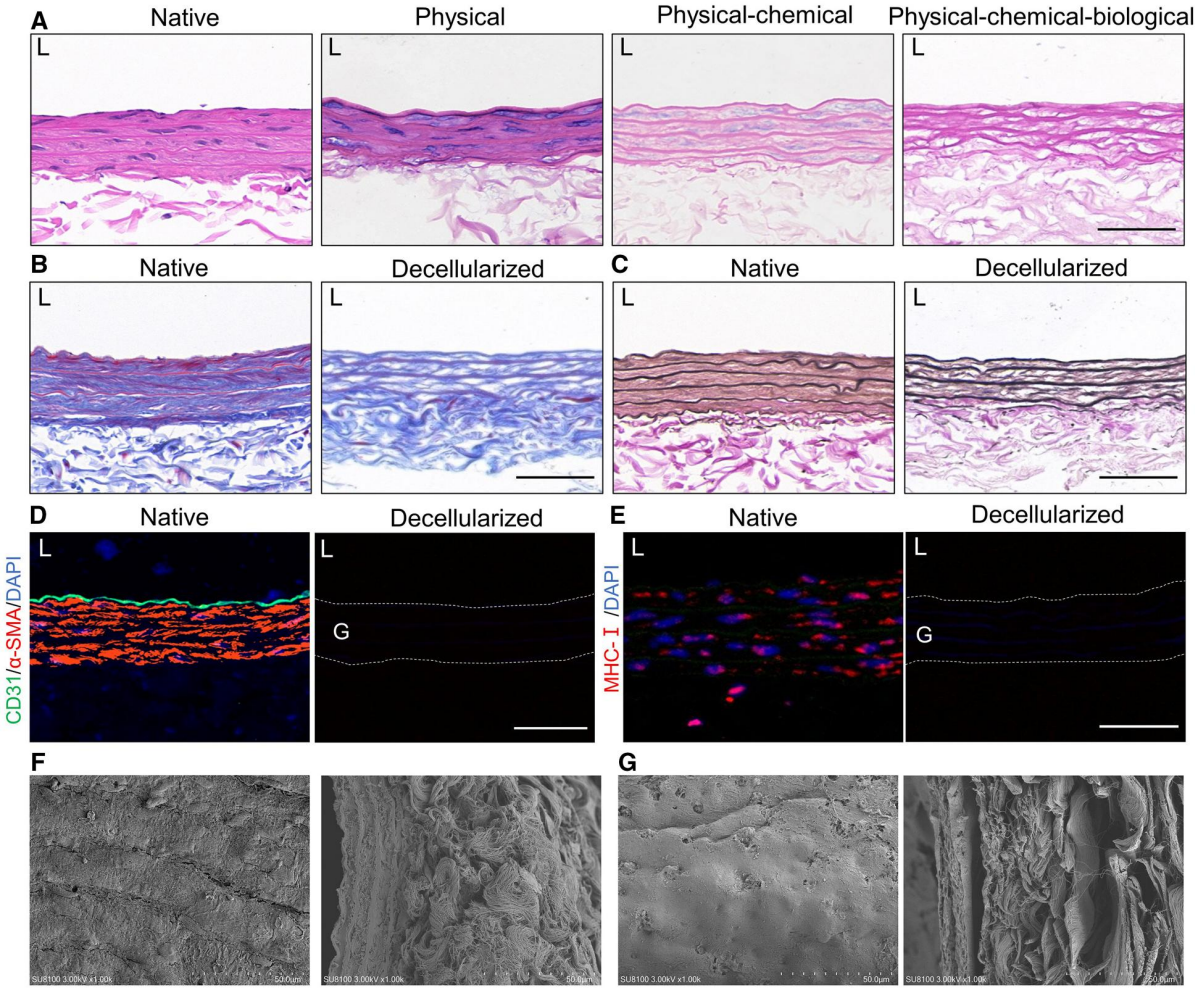
Evaluation of rat carotid artery before and after decellularization. (**A**) Hematoxylin and eosin (HE) staining of the native rat carotid artery and the decellularized rat carotid artery using different decellularization methods. (**B**) Masson’s trichrome staining of the native rat carotid artery and the decellularized rat carotid artery (scale bar, 50 μm). (**C**) Verhoeff’s Van Gieson (EVG) staining of the native rat carotid artery and the decellularized rat carotid artery (scale bar, 50 μm). (**D**) CD31 and α-SMA were eliminated in native and decellularized rat carotid artery (scale bar, 50 μm). (**E**) Major histocompatibility complexes I (MHC-I) was eliminated in native and decellularized rat carotid artery (scale bar, 50 μm). (**F**) Scanning electron microscopy (SEM) evaluations of the internal surface (left) and cross-section (right) of native rat carotid artery. (**G**) SEM evaluations of the internal surface (left) and cross-section (right) of decellularized rat carotid artery.

HE staining demonstrates the absence of cellular structures in the decellularized vessel graft (Figure 3A). There is not much difference between the native rat carotid artery and the decellularized rat carotid artery in terms of gross morphology. When observed under a magnification of 1000 times, the fiber alignment in the decellularized vessel appears more disordered compared to the native vessel. Under the same light source intensity, the decellularized vessel is more transparent (Supplementary Figure S3A and B). Masson’s trichrome staining and EVG staining show good preservation of collagen fibers and elastic fibers in the decellularized graft (Figure 3B and C). Immunofluorescence staining for CD31 and α-SMA confirms the effective removal of cells in the decellularization process (Figure 3D). Staining for major histocompatibility complexes I (MHC-I), essential for the foreign body response, reveals the complete absence of positive cells in the decellularized vessel (Figure 3E). Under scanning electron microscopy (SEM), the external layer of the decellularized vessel shows more irregular fiber fractures and disorganized alignment compared to the native vessel. The internal elastic lamina presents pores and lacks endothelial cell attachment. In cross-section, the decellularized vessels basically retained the structure of natural vessels (Figure 3F and G and Supplementary Figure S3).

### Construction of the allogeneic rat carotid arteriovenous graft model using decellularized vascular graft

**Figure 4.**
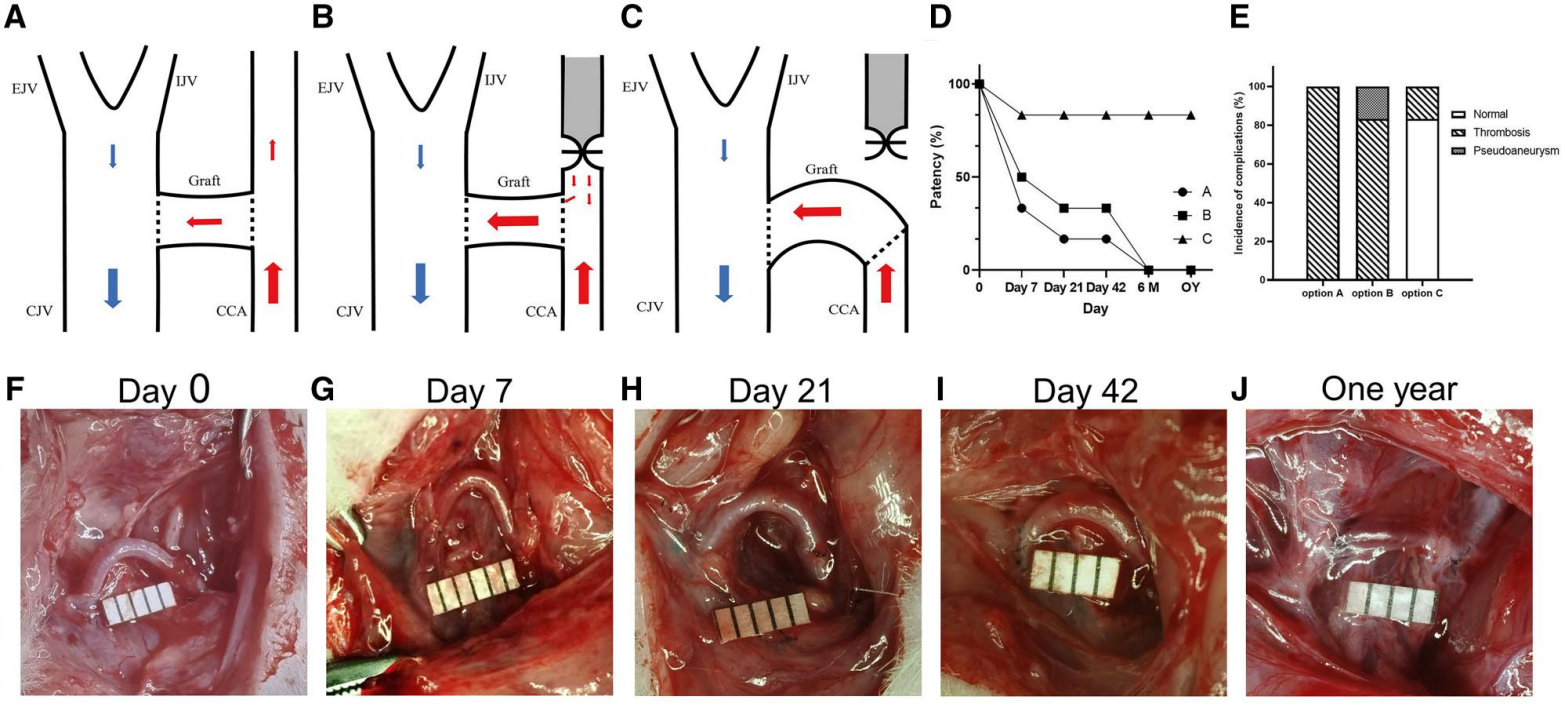
Construction and evaluation of the allogeneic rat carotid arteriovenous graft model using decellularized vascular graft. (**A–C**) Animal model construction using three surgical options. (**D**) Postoperative patency rates of the three different surgical options, with significantly better patency observed in option C compared to option A and option B (*n* = 6). (**E**) Incidence of adverse events in the vascular grafts using three surgical options (*n* = 6). (**F**–**I**) Gross morphology of Day 0 (F), Day 7 (G), Day 21 (H), Day 42 (I) and 1 year (**J**) using option C.

The users of the dialysis access are patients with end-stage CKD. To simulate a similar *in vivo* disease environment, we first established a rat model of renal failure. The details of the renal failure model can be found in Supplementary Figure S4. Initially, we chose option A for model construction, which involved an end-lateral anastomosis of the decellularized vascular graft to both the carotid artery and jugular vein (Figure 4A). However, the patency of the graft was low, and it occluded within a short period of time primarily due to thrombus formation (Figure 4E). We then attempted option B, which included an end-lateral anastomosis of the decellularized vascular graft to both the carotid artery and jugular vein, followed by ligating the proximal end of the carotid artery anastomosis site (Figure 4B). This modification improved graft patency but was still suboptimal, mainly due to thrombus formation at the arterial anastomosis site (Figure 4E). After numerous attempts, we ultimately confirmed option C as the most suitable model construction approach (Figure 4C).

Option C involved the pre-transection of the carotid artery, followed by an end-to-end anastomosis between the decellularized vascular graft and the proximal cut end of the carotid artery. Subsequently, an end-to-side anastomosis was made between the decellularized vascular graft and the jugular vein, positioning the graft as close as possible to the jugular vein to create a ‘semi-circular arc’ appearance, characterized by a gentle and curved alignment resembling half of a circle. This configuration aims to achieve a curvature that is visually akin to a semi-circle, making the anastomosis between the graft and the jugular vein larger for blood flow to pass through. The details of model construction can be found in the ‘Materials and methods’ section. If the model is successful, we would be able to observe prominent pulsations on the surface of the rat’s neck and feel the palpable blood flow pulsations (Supplementary material 2). Option 3 demonstrated the highest short-term and long-term patency rates, with minimal graft thrombosis even without anticoagulation treatment (Figure 4E). Therefore, we used option C in subsequent experiments to construct the animal model. On gross observation, the decellularized vascular grafts showed no significant morphological changes at Day 7, Day 21, Day 42 and one-year post-implantation, maintaining a ‘semi-circular arc’ appearance (Figure 4F–J).

### Assessment of decellularized vascular grafts in a rat carotid arteriovenous graft model using non-invasive ultrasound

**Figure 5.**
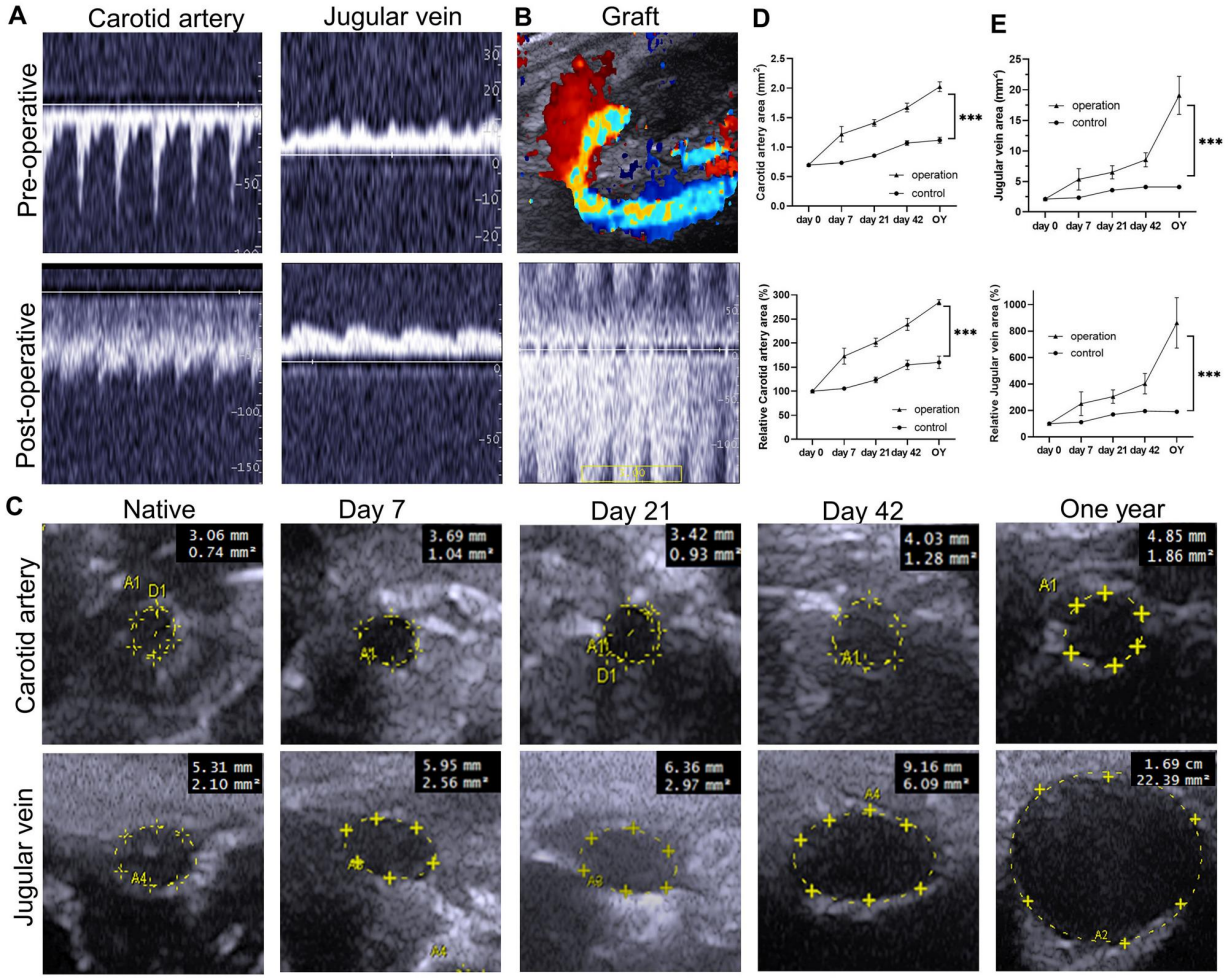
Assessment of decellularized vascular grafts *in vivo* using non-invasive ultrasound. (**A**) Ultrasound spectral analysis of the carotid artery and jugular vein before and after transplantation of decellularized vascular grafts. The appearance of AVG-specific spectral patterns in the carotid artery and jugular vein after transplantation indicates successful modeling. (**B**) Color Doppler blood flow imaging and ultrasound spectrum of decellularized vascular grafts. (**C**) Evaluation of changes in the lumen size of the carotid artery and jugular vein. (**D**) Statistical analysis of the area and relative area of the carotid artery after transplantation (*P* < 0.0001, ANOVA; Day 0, Day 7, Day 21 and Day 42, *n* = 5; 1 year, *n* = 3). (**E**) Statistical analysis of the area and relative area of the jugular vein after transplantation (*P* < 0.0001, ANOVA; Day 0, Day 7, Day 21, and Day 42, *n* = 5; 1 year, *n* = 3).

We evaluated the vascular grafts using non-invasive ultrasound *in vivo*. The appearance of AVG-specific spectral patterns in the carotid artery and jugular vein after transplantation indicates successful modeling (Figure 5A). Color Doppler blood flow imaging reveals the ‘semi-circular arc’ appearance of the vascular grafts *in vivo*, and the ultrasound spectrum of decellularized vascular grafts demonstrates a high blood flow velocity in the graft (Figure 5B). We obtained vascular ultrasound images of the carotid artery and jugular vein at post-transplantation time points of Day 7, Day 21, Day 42 and 1 year, showing dilation of both the carotid artery and jugular vein with prolonged graft survival (Figure 5C–E). *In vivo* ultrasound evaluation of the model exhibits features consistent with clinical AVG outcomes. The results of ultrasound evaluation indicate that the rat carotid arteriovenous graft model using decellularized vascular grafts exhibits features similar to those observed clinically.

### Revascularization of decellularized vascular grafts

**Figure 6.**
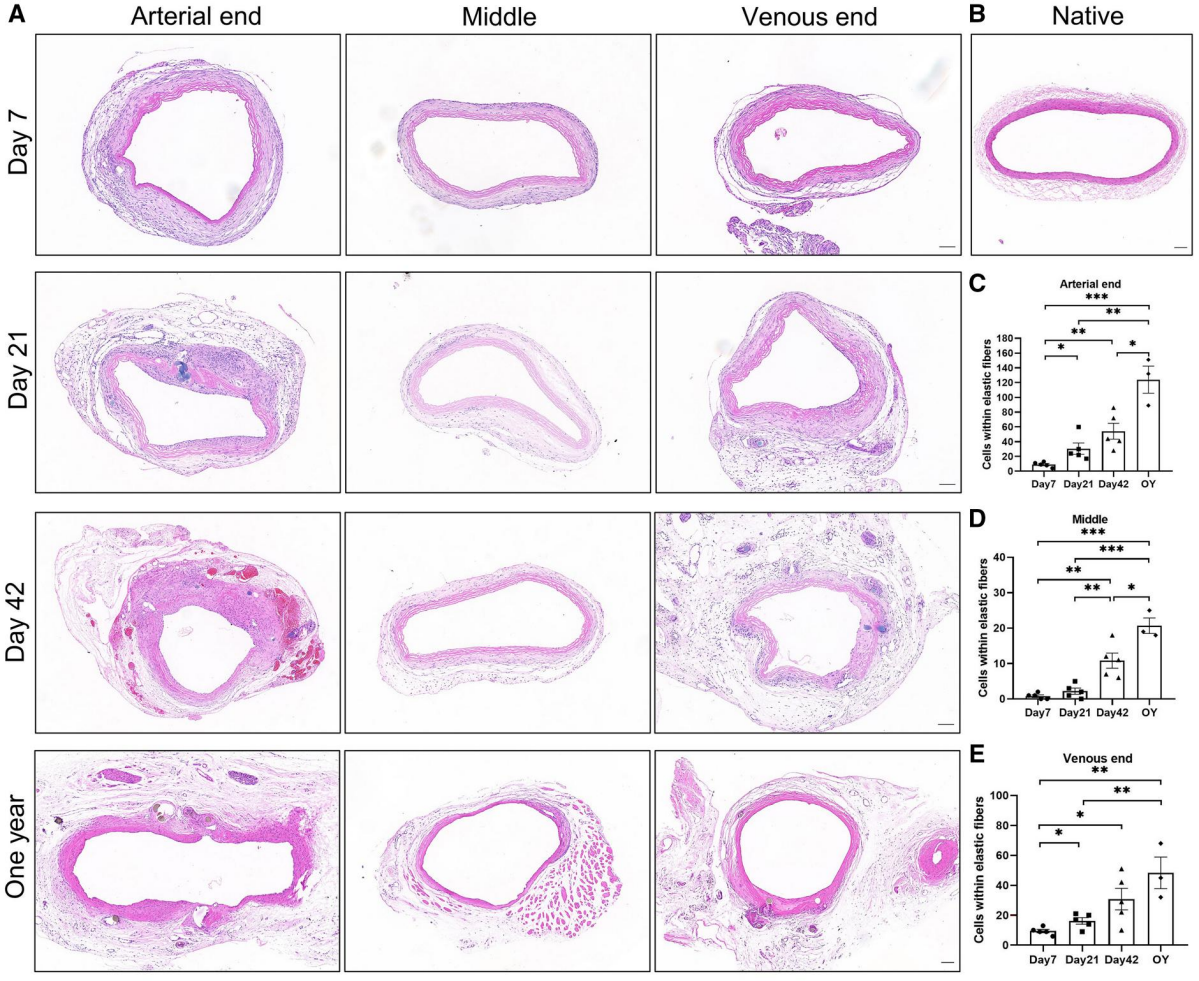
Hematoxylin and eosin (HE) staining of postoperative decellularized vascular grafts. (**A**) HE staining of decellularized vascular grafts at Day 7, Day 21, Day 42, and one-year post-implantation using option C (scale bar, 100 μm). (**B**) HE staining of native rat carotid artery (scale bar, 100 μm). (C–E) Quantification of the number of cells within the elastic fibers of (C) arterial end (*P* < 0.0001, ANOVA; Day 7, Day 21, Day 42, *n* = 5; 1 year, *n* = 3), (D) middle segment (*P* < 0.0001, ANOVA; Day 7, Day 21, Day 42, *n* = 5; 1 year, *n* = 3) and (E) venous end (*P* = 0.0017, ANOVA; Day 7, Day 21, Day 42, *n* = 5; 1 year, *n* = 3).

The revascularization status of decellularized vascular grafts is critical for their functionality. We evaluated the revascularization of the grafts after transplantation. HE staining revealed the histological changes of the decellularized vascular grafts at Day 7, Day 21, Day 42 and 1 year after transplantation. The outer region of the graft exhibited a sufficient thickness of tissue and extensive infiltration of various cell types, resembling the ‘adventitial layer.’ At Day 21, neointima formation was observed at both the arterial and venous ends of the graft, while the middle segment did not show significant intimal hyperplasia. With increasing graft survival time, the number of cells within the elastic fibers increased, and the arterial end of the graft in the one-year group showed a higher cell presence (Figure 6A, C–E). Evaluation of collagen fiber content in the decellularized vascular grafts was performed using Masson’s trichrome staining (Supplementary Figure S5). EVG staining demonstrated relatively well-preserved elastic fibers, and the thickness of the neointima continued to increase (Supplementary Figure S5).

**Figure 7.**
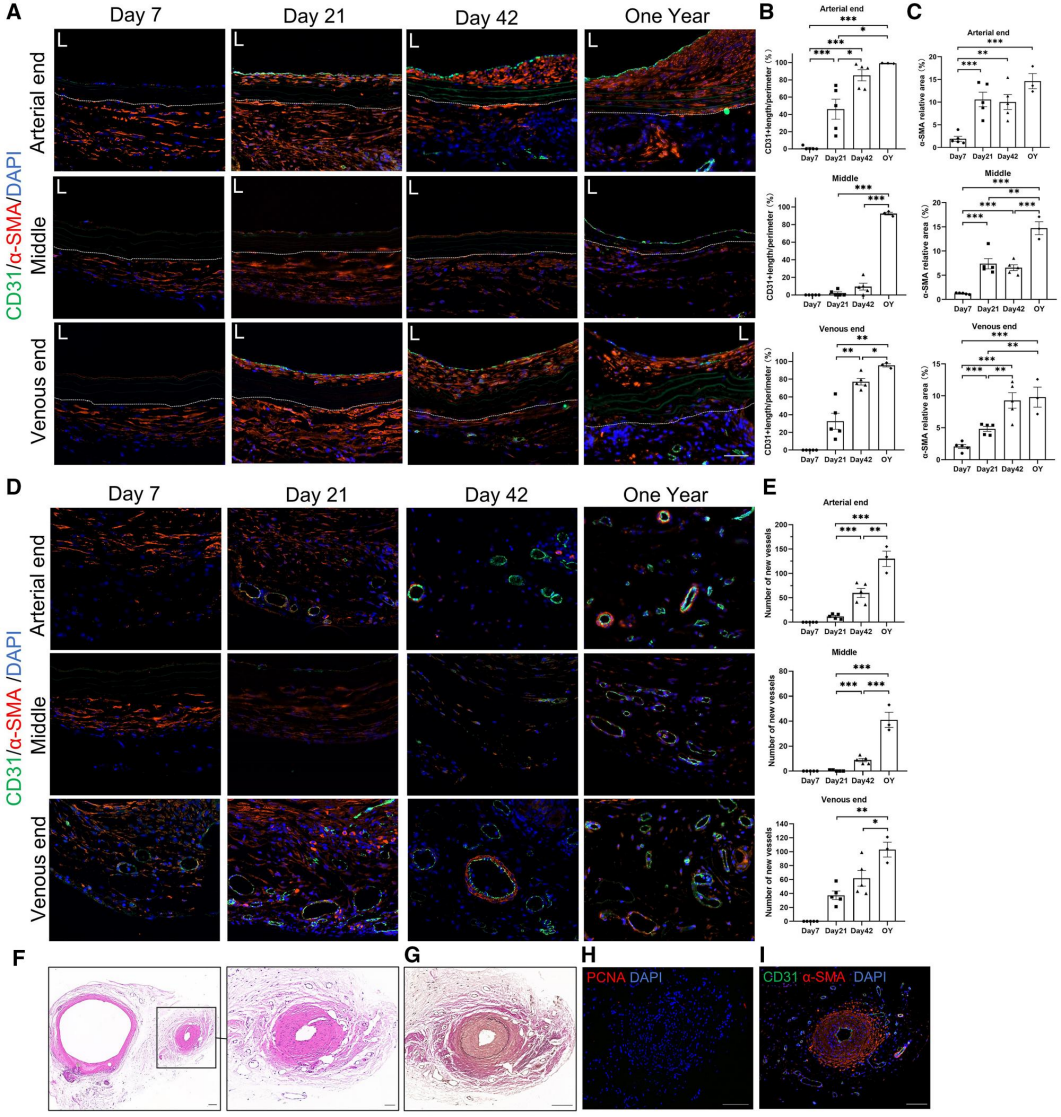
Evaluation of revascularization in decellularized vascular grafts. (**A**) Immunofluorescence staining of CD31 and α-SMA at Day 7, Day 21, Day 42 and 1 year (scale bar, 50 μm). (**B**) Confluence of CD31-positive cells of arterial end (*P* < 0.0001, ANOVA; Day 7, Day 21, Day 42, *n* = 5; 1 year, *n* = 3), middle segment (*P* < 0.0001, ANOVA; Day 7, Day 21, Day 42, *n* = 5; 1 year, *n* = 3) and venous end (*P* < 0.0001, ANOVA; Day 7, Day 21, Day 42, *n* = 5; 1 year, *n* = 3). (**C**) α-SMA-positive area of arterial end (*P* < 0.0001, ANOVA; Day 7, Day 21, Day 42, *n* = 5; 1 year, *n* = 3), middle segment (*P* < 0.0001, ANOVA; Day 7, Day 21, Day 42, *n* = 5; 1 year, *n* = 3) and venous end (*P* < 0.0001, ANOVA; Day 7, Day 21, Day 42, *n* = 5; 1 year, *n* = 3). Because the region of the vascular adventitia lacks precise boundaries, only the intimal and medial layers of the blood vessel are taken into account. (**D**) Neovascularization in the periphery of decellularized vascular grafts, with CD31-positive cells and α-SMA-positive cells (scale bar, 50 μm). (**E**) Number of neovessels in the periphery of decellularized vascular grafts at arterial end (*P* < 0.0001, ANOVA; Day 7, Day 21, Day 42, *n* = 5; 1 year, *n* = 3), middle segment (*P* < 0.0001, ANOVA; Day 7, Day 21, Day 42, *n* = 5; 1 year, *n* = 3) and venous end (*P* < 0.0001, ANOVA; Day 7, Day 21, Day 42, *n* = 5; 1 year, *n* = 3), (*P* < 0.0001 , Day 7, Day 21, Day 42, *n* = 5; 1 year, *n* = 3). (**F–I**) Newly formed vessel with intimal hyperplasia were observed at the venous end of the vascular grafts in the one-year group, hematoxylin and eosin staining (F), Verhoeff’s Van Gieson staining (G), immunofluorescence staining of PCNA (H) and immunofluorescence staining of CD31 and α-SMA (I) (scale bar, 50 μm). The area outside the dashed lines represents the outer of decellularized vascular grafts.

We evaluated the recellularization of decellularized vascular grafts at Day 7, Day 21, Day 42 and 1 year after transplantation using immunofluorescence (Figure 7). At Day 7, adherent cells were observed on the luminal surface of the vascular grafts. By Day 21, the luminal surfaces of both the arterial and venous ends of the grafts were mostly covered with CD31-positive cells, while the middle segment did not show CD31-positive cell presence until Day 42, indicating earlier endothelialization at the arterial and venous ends compared to the middle segment. After 1 year, the middle segment of the grafts was mostly covered with CD31-positive cells, indicating completion of endothelialization throughout the entire graft (Figure 7A and B). The neointima of the grafts continued to thicken, resulting in an increase of α-SMA-positive area. The α-SMA-positive area within the grafts also increased, and a significant number of α-SMA-positive cells were found in the elastic fibers of the arterial end in the one-year group, indicating successful recellularization at the arterial end which is superior to the venous end and middle segment (Figure 7A and C).

Additionally, we observed significant neovascularization in the periphery of the vascular grafts, and we assessed the growth of neovessels in the peripheral region of the grafts (Figure 7D and E). Neovessels appeared earliest at the venous end of the grafts, with CD31 and α-SMA dual-positive neovessels detected as early as Day 7. Neovessels were observed at the arterial end of the grafts on Day 21. Neovessels in the middle segment of the grafts appeared later, with only a few neovessels observed at Day 42. Regardless of the arterial end, venous end or middle segment of the grafts, the number of neovessels continued to increase within 1 year. Interestingly, in the venous end of the grafts in the one-year group, we discovered a neovessel that exhibited significant intimal hyperplasia (Figure 7F–I), this neovessel already displayed the presence of new elastic fibers within its structure.

### Cell proliferation and apoptosis in the decellularized grafts and surrounding tissues after transplantation

**Figure 8.**
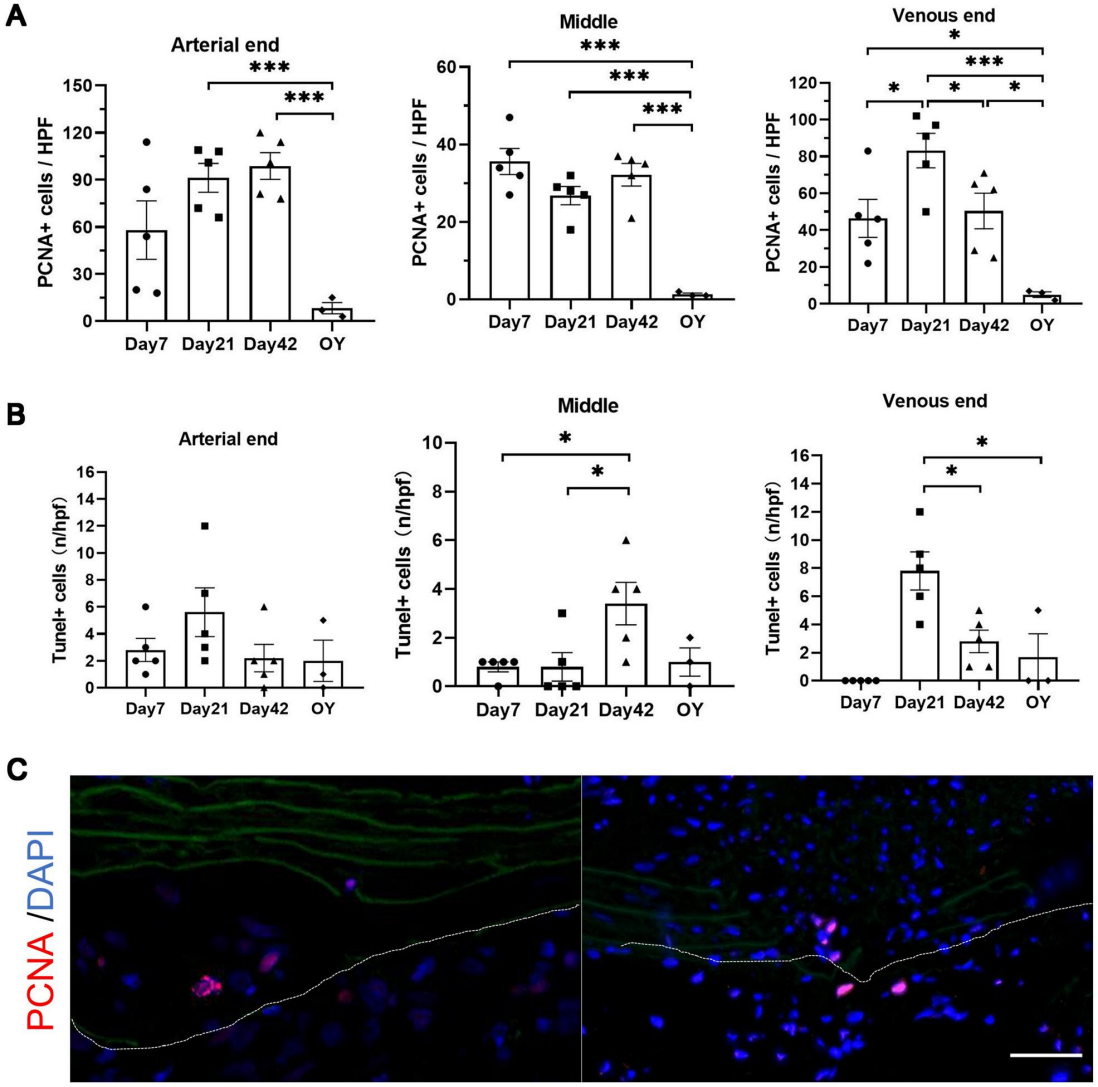
Evaluation of the proliferation and apoptosis status of cells in the decellularized vascular grafts and surrounding tissues. (**A**) Statistical analysis of PCNA-positive cells of arterial end (*P* = 0.0018, ANOVA; Day 7, Day 21, Day 42, *n* = 5; 1 year, *n* = 3), middle segment (*P* < 0.0001, ANOVA; Day 7, Day 21, Day 42, *n* = 5; 1 year, *n* = 3) and venous end (*P* = 0.0012, ANOVA; Day 7, Day 21, Day 42, *n* = 5; 1 year, *n* = 3). (**B**) Statistical analysis of Tunel-positive cells of arterial end (*P* = 0.2391, ANOVA; Day 7, Day 21, Day 42, *n* = 5; 1 year, *n* = 3), middle segment (*P* = 0.0215, ANOVA; Day 7, Day 21, Day 42, *n* = 5; 1 year, *n* = 3) and venous end (*P* = 0.0004, ANOVA; Day 7, Day 21, Day 42, *n* = 5; 1 year, *n* = 3). (**C**) PCNA-positive cells at the site of elastic fiber fractures (scale bar, 50 μm). The area outside the dashed lines represents the outer of decellularized vascular grafts.

We evaluated the immunofluorescence staining of PCNA in the decellularized vascular grafts after transplantation (Figure 8A and Supplementary Figure S6A). At Day 7, PCNA-positive cells were mainly concentrated around the decellularized vascular grafts. By Day 21, PCNA-positive cells were observed within the elastic fibers of both the arterial and venous ends of the vascular grafts. PCNA-positive cells were also present in the neointima of the arterial and venous ends at Day 21. At Day 42, the number of PCNA-positive cells increased within the elastic fibers and neointima of the vascular grafts. Interestingly, at 1 year, there were few PCNA-positive cells observed in both the arterial and venous ends as well as the middle segment of the vascular grafts, almost no positive cells were detected. Furthermore, we observed that there appeared to be a higher accumulation of PCNA-positive cells at the sites of elastic fiber rupture in the vascular grafts (Figure 8C). We evaluated the apoptosis status after transplantation of decellularized vascular grafts using Tunel staining (Figure 8B, Supplementary Figure S6C). The results indicated that apoptosis was not significantly prominent in the decellularized vascular grafts and surrounding tissues after transplantation.

### Infiltration of CD68-positive cell and CD3-positive cell in the decellularized vascular grafts and surrounding tissues after transplantation

**Figure 9.**
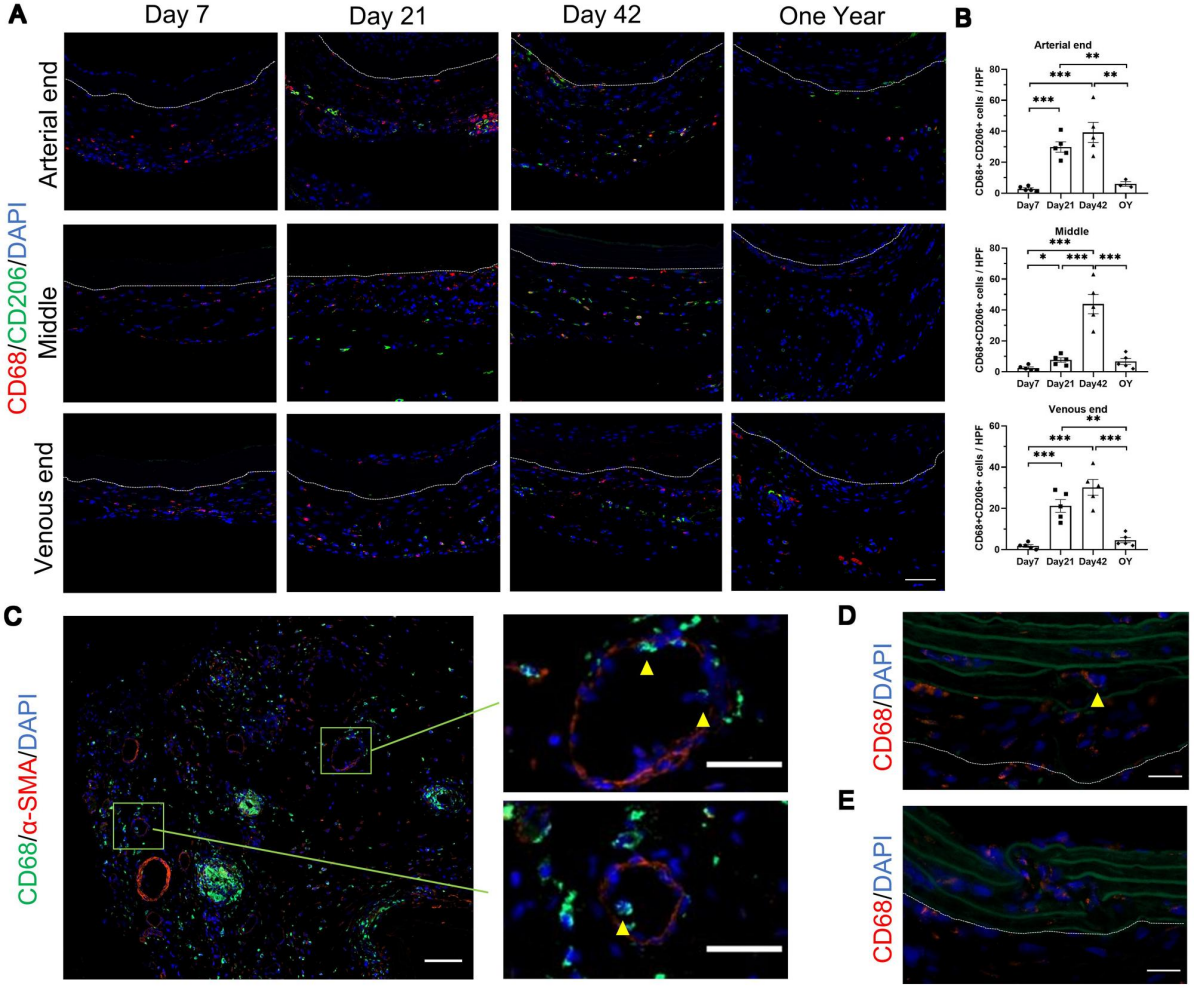
Accumulation of macrophages around the decellular vascular graft. (**A**) M2 macrophages around the decellular vascular graft (scale bar, 50 μm). (**B**) Statistical analysis of M2 macrophages of arterial end (*P* < 0.0001, ANOVA; Day 7, Day 21, Day 42, *n* = 5; 1 year, *n* = 3), middle segment (*P* < 0.0001, ANOVA; Day 7, Day 21, Day 42, *n* = 5; 1 year, *n* = 3) and venous end (*P* < 0.0001, ANOVA; Day 7, Day 21, Day 42, *n* = 5; 1 year, *n* = 3). (**C**) Presence of CD68-positive cells in α-SMA-positive neovessels (scale bar, 50 μm). (**D**) CD68-positive cells at the site of disrupted elastic fibers, arrows show fractured and small elastic fibers around the CD68-positive cell (scale bar, 20 μm). (**E**) Increased number of CD68-positive cells at the site of disrupted elastic fibers, green represents elastic fibers (scale bar, 20 μm). The area outside the dashed lines represents the outer of decellularized vascular grafts.

Macrophages mediate inflammatory responses and tissue repair. They can generally be roughly classified into M1 macrophages and M2 macrophages. M1 macrophages mediate early inflammatory responses, promoting a pro-inflammatory phenotype. M2 macrophages are primarily involved in tissue repair, exhibiting an anti-inflammatory phenotype. We evaluated the infiltration of M1 macrophages (Supplementary Figure S7) and M2 macrophages (Figure 9A and B) of decellularized vascular graft after transplantation. CD68-positive cells predominantly infiltrated the periphery of the graft at Day 7, with infiltration observed throughout the entire length of the graft. Significant infiltration was still present at Day 21 and Day 42, with CD68-positive cells observed within the elastic fiber layer. In the one-year group, fewer CD68-positive cells were observed, indicating reduced macrophage infiltration within the graft at this stage. CD68-positive cells were rarely observed in the neointima in most samples. We observed that the infiltration of macrophages after Day 7 was mainly dominated by M2 macrophages (CD68 and CD206 double-positive cells), and there was increased infiltration at Day 21 and Day 42 (Figure 9A). This reflects the *in vivo* repair process of decellularized grafts during this period. In contrast, infiltration of M1 macrophages (CD68 and iNOS double-positive cells) was scarce after Day 7 (Supplementary Figure S7). Furthermore, we performed immunofluorescence of α-SMA and CD68 on the venous end of the graft at Day 42. Interestingly, we found that neovessels served as one of the pathways for macrophage infiltration around the graft periphery (Figure 9C). Additionally, we observed clusters of macrophage infiltration around the periphery of the graft, some of which were localized around suture lines (Figure 9C). We discovered an increased number of CD68-positive cells at sites of fractured elastic fibers within the graft. Fractured and small elastic fibers were observed surrounding CD68-positive cells (Figure 9D and E).

**Figure 10.**
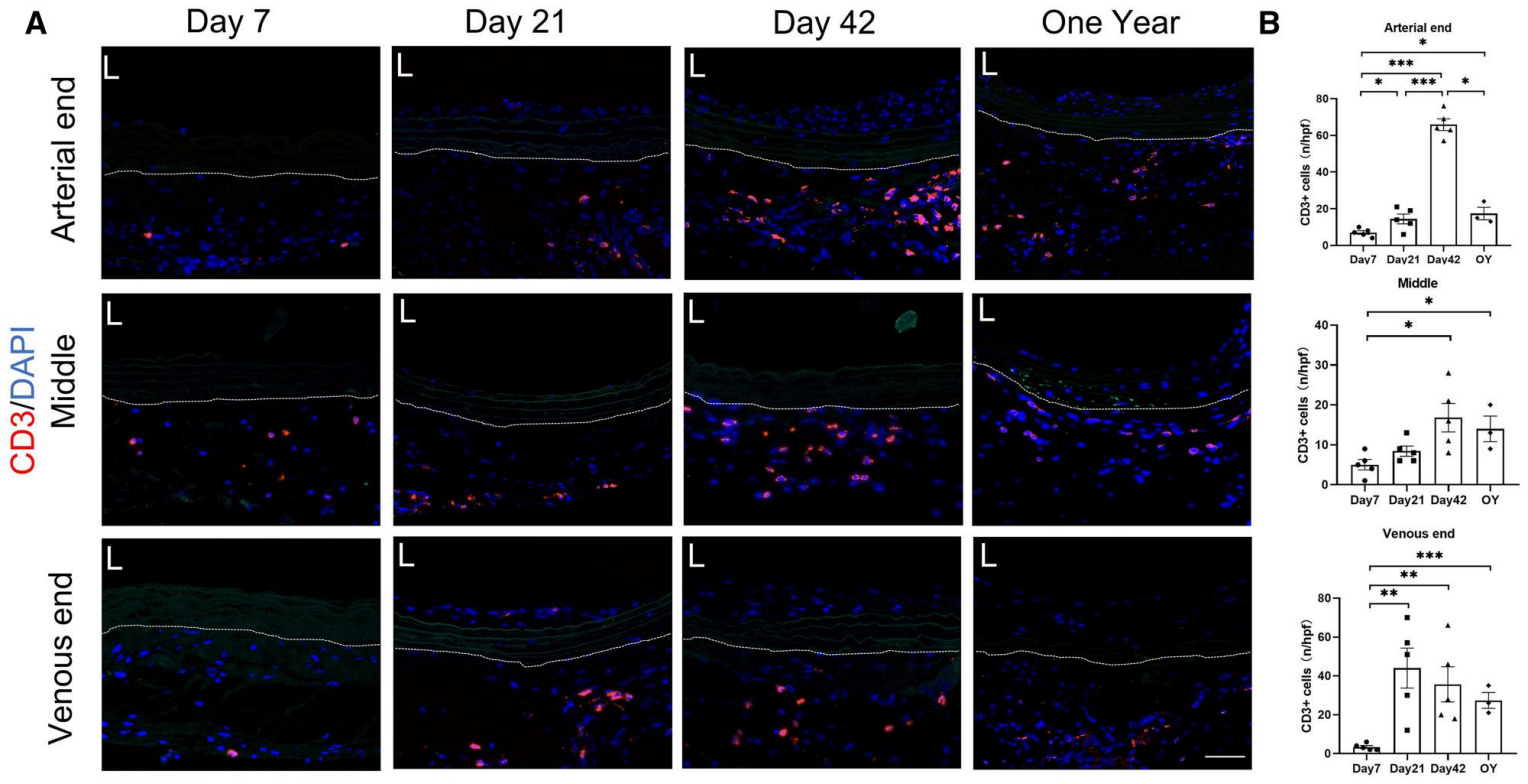
Accumulation of CD3-positive cells around the decellular vascular graft. (**A**) CD3-positive cells around the decellular vascular grafts (scale bar, 50 μm). (**B**) Statistical analysis of CD3-positive cells of arterial end (*P* < 0.0001, ANOVA; Day 7, Day 21, Day 42, *n* = 5; 1 year, *n* = 3), middle segment (*P* = 0.0151, ANOVA; Day 7, Day 21, Day 42, *n* = 5; 1 year, *n* = 3) and venous end (*P* = 0.0102, ANOVA; Day 7, Day 21, Day 42, *n* = 5; 1 year, *n* = 3). The area outside the dashed lines represents the outer of decellularized vascular grafts.

T lymphocytes play a crucial role in organ transplant rejection, generally mediating the acute phase of immune rejection. Therefore, their involvement in the short-term survival of decellularized vascular grafts is essential. We evaluated the infiltration of CD3-positive cells (T cells) at Day 7, Day 21, Day 42 and 1 year after transplantation (Figure 10). We found that the infiltration of CD3-positive cells around the graft was not significant at Day 7, indicating that the acute phase immune rejection response against the graft was not severe. Interestingly, with increasing graft survival time, we observed greater infiltration of CD3-positive cells around the graft, with a higher number of CD3-positive cells observed in the one-year group (Figure 10). This suggests that the influence of CD3-positive cells (T cells) on the graft may extend beyond the acute phase of immune rejection.

## Discussion

Decellularized vascular grafts have demonstrated promising patency rates and biocompatibility in some clinical trials [10]. However, the understanding of their *in vivo* adaptation remains limited. Due to the lack of suitable small animal models, the mechanisms and signaling pathways involved in the *in vivo* adaptation of decellularized vascular grafts are unknown. As a result, targeted investigations into the grafts’ adaptation processes and their interactions with cells and tissues in the body are currently unavailable. This study evaluated the histological condition of allogeneic decellularized vascular grafts used as hemodialysis conduits in the human body 5 years after transplantation. Subsequently, we established a small animal model in rats with similar histological and hemodynamic characteristics to human. Furthermore, we undertook a pioneering investigation into the *in vivo* adaptation process of decellularized blood vessels within a small animal model. We found that: (i) The adaptation of decellularized vascular grafts in the human body after 5 years of transplantation is considered ideal. (ii) The rat neck AVG model constructed using decellularized vascular grafts effectively replicated the histological and hemodynamic characteristics observed in the human body after transplantation of decellularized vascular grafts. (iii) The abundant neovessels surrounding the graft and the influence of elastic fiber structure on the recellularization of decellularized vascular grafts deserve attention. (iv) Macrophage infiltration was primarily concentrated in the early post-transplantation period, while T cell infiltration increased continuously within 1 year after transplantation.

We found that the adaptation of allogeneic decellularized vascular grafts in the human body was ideal. In blood vessels, α-smooth muscle actin (α-SMA) positive cells are typically associated with smooth muscle cells and play crucial roles in supporting and regulating vascular functions such as maintenance of vascular structure [23], response to injury [24], and regulation of vascular tension [25]. Five years after transplantation, the transplanted graft showed the presence of α-SMA-positive spindle-shaped cells in the interior (Figure 1). This indicates that the transplanted graft has been infiltrated by smooth muscle-like cells, and suggests that the cellularization of the decellularized graft is promising and at least partially capable of functioning like normal blood vessels. However, further experiments are needed to verify if the decellularized graft can possess functions like vasoconstriction and vasodilation similar to normal blood vessels. Even after 5 years of use, the decellularized graft’s structure remains intact, with the extracellular matrix components fully preserved, and no evidence of fibrous capsule encapsulating the graft (Figure 1A). Fibrous capsule formation is considered a biomaterial-mediated foreign body reaction, where biomaterials become encapsulated within fibrous tissue and isolated from the surrounding tissues, which can significantly impede graft recellularization [26]. This suggests good biocompatibility of the allogeneic decellularized vascular graft. Additionally, we observed an increased number of neovessels in the periphery of the transplanted graft (Figure 1B). These neovessels are believed to provide nutrients to the graft [27, 28]. Both the transplanted graft and AVF are exposed to a renal failure microenvironment, and the transplanted graft shows more infiltration of macrophages and T cells compared to AVF (Figure 1D and E), possibly due to chronic inflammatory responses triggered by the graft implantation. These inflammatory responses may contribute to the higher number of neovessels observed in the periphery of the graft, but further research is needed to confirm this hypothesis. Non-invasive ultrasound evaluation revealed that the anastomotic site of the transplanted graft exhibited intimal hyperplasia similar to that observed in AVF (Figure 2G–I). Intimal hyperplasia in AVF is associated with endothelial–mesenchymal transition, smooth muscle cell proliferation, migration, and phenotypic switching [29]. Interestingly, early-stage transplanted graft lacked smooth muscle cells, yet exhibited similar intimal hyperplasia (especially evident in animal models). This suggests that the mechanisms underlying intimal hyperplasia in decellularized vascular grafts might not be identical to those in AVF, warranting further investigation to identify targeted solutions.

Previous studies predominantly utilized large animals such as pigs, cows and dogs, lacking suitable small animal models. This limitation hindered the exploration of the advantages of small animal models in life sciences and medical research for investigating the biological processes of decellularized vessels *in vivo*. Our model showed a high patency rate in the early stage (Figure 4D), and the patency rates at 21 days and 1 year were comparable. This indicates good *in vivo* adaptation of the decellularized vascular grafts. Non-invasive ultrasound imaging showed that our model exhibited features similar to clinical cases, such as changes in hemodynamics [30, 31] and vascular morphology [2]. By measuring the area change within the elastic lamina of the graft in HE staining, we found that the graft did not undergo expansion in the early post-transplantation period and only exhibited mild enlargement at arterial and venous ends after 1 year. This suggests that the mechanical properties of the decellularized vascular graft are sufficient to withstand blood flow impact, similar to the current clinical application of decellularized vascular grafts [10, 13].

Previous studies on artificial blood vessels have focused more on the cellularization within the grafts [32, 33], or the endothelialization and intimal hyperplasia on the luminal surface [34, 35], paying less attention to neovascularization surrounding the grafts. Vascular networks play a crucial role in transporting blood, delivering oxygen and providing nutrients to tissues at the microvasculature interface. Therefore, the formation of vessels at the microcirculatory level is essential for tissue regeneration and repair [36]. We observed a significant amount of neovessels around the decellularized vascular grafts. Interestingly, at the venous end in the one-year group, we discovered a neovessel exhibiting significant intimal hyperplasia (Figure 7F–I), similar to the intimal hyperplasia seen in AVF. This suggests that high-velocity blood flow from the artery directly enters this newly formed blood vessel. Furthermore, we detected CD68-positive cells within and around the neovessels, indicating that macrophages in the blood may quickly infiltrate the area around the graft through the neovessels. Interestingly, a recent study also found the accumulation of macrophages (F4/80-positive cells) around newly formed vessels in a mouse hindlimb ischemia model [37]. It is evident that immune cells play a regulatory role in neovascularization. For instance, vascular endothelial growth factor (VEGF) released from M1 or M2 cells directly regulates endothelial cell proliferation [38]. Additionally, direct interactions of monocytes/macrophages with endothelial cells may stimulate endothelial proliferation and mediate the fusion of endothelial tip cell [39, 40]. However, further research is needed to determine whether the generation of these new vessels accelerates the infiltration of immune cells.

The elastic fiber layer is the main structural component of the vascular media, composed of tightly connected elastic proteins, which requires specific arrangement of smooth muscle cells to form [41, 42]. Previous studies have suggested that the elastin lamina in decellularized tissues prevents cell infiltration and suppresses recellularization [43]. We also observed a large number of α-SMA-positive cells filling the elastic fiber layer of the one-year group, indicating that the inhibitory effect of the elastic fiber layer on cell infiltration is not absolute. Decellularization processes can cause certain damage to the elastic fiber layer (Figure 9C and D), and we found that PCNA-positive cells tend to aggregate at the rupture sites of the elastic fibers and show a tendency to proliferate and migrate into the elastic fiber layer (Figure 8C). This suggests that cells can proliferate and migrate inward along the sites of damage in the elastic fiber layer. Decellularized vascular grafts have demonstrated advantages in clinical trials, and by modifying the elastic fiber layer of decellularized vascular graft to facilitate recellularization without compromising mechanical performance, the application prospects of decellularized vascular grafts can be greatly improved.

Due to the lack of suitable and available small animal models for AVG using decellularized vascular grafts, there have been limited research specifically reporting changes in the infiltration of immune cells after decellularized vascular graft transplantation. In our constructed the allogeneic rat carotid arteriovenous graft model using decellularized vascular graft, we evaluated the infiltration of macrophages and T cells. Macrophages are an important type of immune cell involved in inflammatory reactions, producing growth factors and cytokines, and regulating the activities of other cell types [44]. Understanding the role of macrophages in the readaptation process of decellularized vascular grafts is crucial. We found that macrophages began to infiltrate around the grafts in the early stages after transplantation (Figure 9A), while fewer macrophages were observed in the one-year group. As a foreign tissue, grafts elicit a response from the immune system [13, 45], and macrophages around the grafts may be involved in the early occurrence of inflammatory reactions. Furthermore, previous studies have shown that the interaction between macrophages and vascular smooth muscle cells may play an important role in vascular wall reconstruction and structural stability [44]. We observed that the infiltration of macrophages after Day 7 was mainly dominated by M2 macrophages. M2 macrophages can also secrete growth factors and cytokines, such as VEGF, which have been shown to regulate neovascular formation [46]. Additionally, we found macrophage infiltration at the rupture sites of the elastic fiber layer (Figure 9D and E), indicating that macrophages may affect the degradation of the elastic fiber layer. Further research is needed to clarify the exact role of macrophage infiltration in the early stages after decellularized vascular graft transplantation and to provide potential strategies for improving graft survival and revascularization.

T cells play a crucial role in the acute rejection of organ transplantation. It is generally believed that T cell-mediated immune rejection is one of the main mechanisms, especially in the early stages after transplantation, typically within days to weeks [47]. In our model, we found that the infiltration of CD3-positive cells in the early stages after transplantation was not significant, indicating that decellularization indeed reduces the host’s rejection response to decellularized vascular grafts. Interestingly, Interestingly, even in grafts transplanted after 1 year, we observed a significant infiltration of CD3-positive cells. The investigation of T lymphocyte response to synthetic biomaterials *in vivo* also observed an increase in T cell numbers after transplantation (after Day 14) [48]. Another study revealed that circulating T lymphocytes could be attracted and activated by cytokines released from biomaterial-adherent macrophages [49]. T lymphocytes play significant roles in orchestrating cell-mediated immune responses and prior investigations have presented evidence of T lymphocytes actively participating in mediating both normal and pathophysiologic events associated with vasculature [50, 51]. Those suggest that T cells in the vascular grafts may have additional roles beyond immune rejection. Currently, we do not have a clear understanding of the subtypes and specific roles of these T cells, but there is no doubt that the role of T cells after decellularized vascular graft transplantation warrants further investigation.

## Conclusion

Decellularized vascular grafts have been proven feasible for constructing dialysis access pathways in clinical trials. However, current research mainly focuses on large animal models and clinical trials, and there is a lack of small animal models that can be used for mechanistic studies. We developed a same-species allogeneic rat model of decellularized vascular graft grafts in the carotid artery, which mimics clinical characteristics. This small animal model provides a foundation for in-depth studies on the adaptation process and improvement strategies of decellularized vascular graft grafts *in vivo*. In this article, we firstly evaluated the histological condition, patency and intimal hyperplasia of decellularized vascular grafts in the human body 5 years after transplantation. In order to replicate an animal model with similar histological and hemodynamic characteristics, we utilized allogeneic decellularized vascular grafts to construct a rat carotid arteriovenous graft model. We provide detailed descriptions of the surgical procedure for model construction, the process of revascularization, the characteristics of neovascularization around the graft, the impact of the elastic fiber layer on graft recellularization, and the infiltration characteristics of macrophages and T cells at different time points in the graft.

## Supplementary Material

rbae029_Supplementary_Data

## Data Availability

Data can be provided upon request.
