# Supplementary material for: Adaptation process of decellularized vascular grafts as hemodialysis access in vivo
Source: Regen Biomater. 2024 Mar 21;11:rbae029. doi: 10.1093/rb/rbae029 (PMC11026144; doi:10.1093/rb/rbae029)
Supplement: rbae029_Supplementary_Data [file rbae029_supplementary_data.zip › supplementary material 1.docx]

Supplementary materials

Method

***Instruments and Reagents***

Operative microscope (Leica Microsystems, cat. no. M125), medical Tape (3M）, tweezers with teeth (Fine Science Tools），micro scissors（Fine Science Tools），micro curved forceps (Fine Science Tools)，micro straight forceps (Fine Science Tools)，microsurgical clamps（Shanghai Jinzhong, W40080）, Isoflurane (RWD china)，Small animal gas anesthesia machine (RWD china)，3-0 silk suture (Surgical Specialties), 8–0 sterile microsuture (Surgical Specialties), 11–0 sterile nylon suture with threaded needle black polyamide monofilament non-absorbable (Aros Surgical Instruments), Ultrasound for small animals (Vevo®3100 LT)，Hair removal cream (Ardell Surgi-Cream), Iodophor cotton balls, saline.

***Surgical Procedure***

The Rat Carotid Artery-Jugular Vein AVG model requires technical skills and microvascular surgical expertise. **We recommend having at least one trained surgeon perform the surgical procedures**.

(1) Anesthesia: The SD rats were anesthetized using isoflurane and maintained under anesthesia throughout the surgery. (**Continuous monitoring of heart and respiratory rates is crucial, as unsuccessful anesthesia can be a significant factor leading to model mortality.**)

(2) Hair preparation: The hair over the neck area was shaved using an electric hair clipper and hair removal cream. Afterward, the area was cleaned with saline to remove any remaining hair removal cream.

(3) Disinfection: The shaved area was disinfected with iodine solution using cotton balls. Sterile drapes were then placed over the prepared site.

(4) Incision: Make a midline incision of approximately 1.5-2.0 cm on the neck. Using dissecting forceps (with a tip diameter of 0.30 mm), bluntly dissect the subcutaneous and adipose tissues layer by layer.

(5) Dissection of the jugular Vein: Observe the shaved area on the rat's neck where two pulsating points symmetrically mark the junction of the sternocleidomastoid muscle and the clavicular part of the pectoral muscle on both sides. The location deep within this area corresponds to the jugular vein. Locate the pulsating point on the right side and continue the dissection using blunt dissecting forceps to separate the connective tissue layer by layer until the jugular vein (a deep purple-colored vessel) is visible. Use dissecting forceps (with a tip diameter of 0.15 mm) to dissect the jugular vein.

(6) Removal of the sternocleidomastoid muscle: The rat's carotid artery is located deep within the sternocleidomastoid muscle. Removing this muscle facilitates the transplantation of decellularized blood vessels. Ligature the ends of the sternocleidomastoid muscle using 3-0 silk sutures, ensuring a long distance between the ligature points on both sides (at least 1.0 cm or more) to provide enough surgical operating space. The purpose of ligating is to prevent minor vessel bleeding within the muscle after the sternocleidomastoid muscle is cut.

(7) Dissection of the carotid artery: After removing the sternocleidomastoid muscle, a strongly pulsating vessel becomes visible. Using micro forceps (with a tip diameter of 0.15 mm), gently separate the overlying muscle and fascial tissues to reveal a distinct vascular sheath structure. Within the vascular sheath, there is a pink, strongly pulsating vessel, which is the carotid artery. Adjacent to the carotid artery is a slightly thinner white tissue, which is the vagus nerve. It is essential not to pull or damage the vagus nerve during the surgery, as it can lead to the animal's death. Continue using micro dissecting forceps to isolate a segment of the carotid artery (at least 1.0 cm in length).

(8) Ligature of the distal end of the carotid artery: Use an 8-0 nylon suture to ligate the distal end of the carotid artery.

(9) Vascular occlusion clamp was used to block the proximal blood flow of the carotid artery. A 1.5mm longitudinal incision was made in the carotid artery, and a decellularized vascular graft was trimmed at a 45° angle. The carotid artery and decellularized blood vessel were anastomosed end-to-side using interrupted sutures with 11-0 vascular thread, typically requiring 8 stitches. The suturing procedure took approximately 15 minutes.

(10) Vascular occlusion clamp was used to block the proximal and distal blood flow of the jugular vein. A 2mm longitudinal incision was made in the jugular vein, and a decellularized vascular graft was trimmed at a 60° angle. The jugular vein and decellularized blood vessel were anastomosed end-to-side using interrupted sutures with 11-0 vascular thread, typically requiring 12 stitches. The suturing procedure took approximately 20 minutes.

(11) When releasing the occlusion clamp on the jugular vein, it was observed that the venous blood flow was smooth, with a small amount of blood flowing back into the decellularized vascular graft. Upon releasing the proximal occlusion clamp of the carotid artery, a rapid filling of blood flow was observed in both the decellularized vascular graft and jugular vein. Generally, there was a slight bleeding at the arterial anastomotic site, which was then controlled by using a microvascular occlusion clamp to occlude the proximal carotid artery for 2 minutes to achieve hemostasis.

(12) Finally, all occlusion clamp were released, and it was observed that the blood vessels pulsated well, with significant dilation at the proximal end of the jugular vein, and no leakage at the anastomotic site. The surgical incision was closed layer by layer.

Results


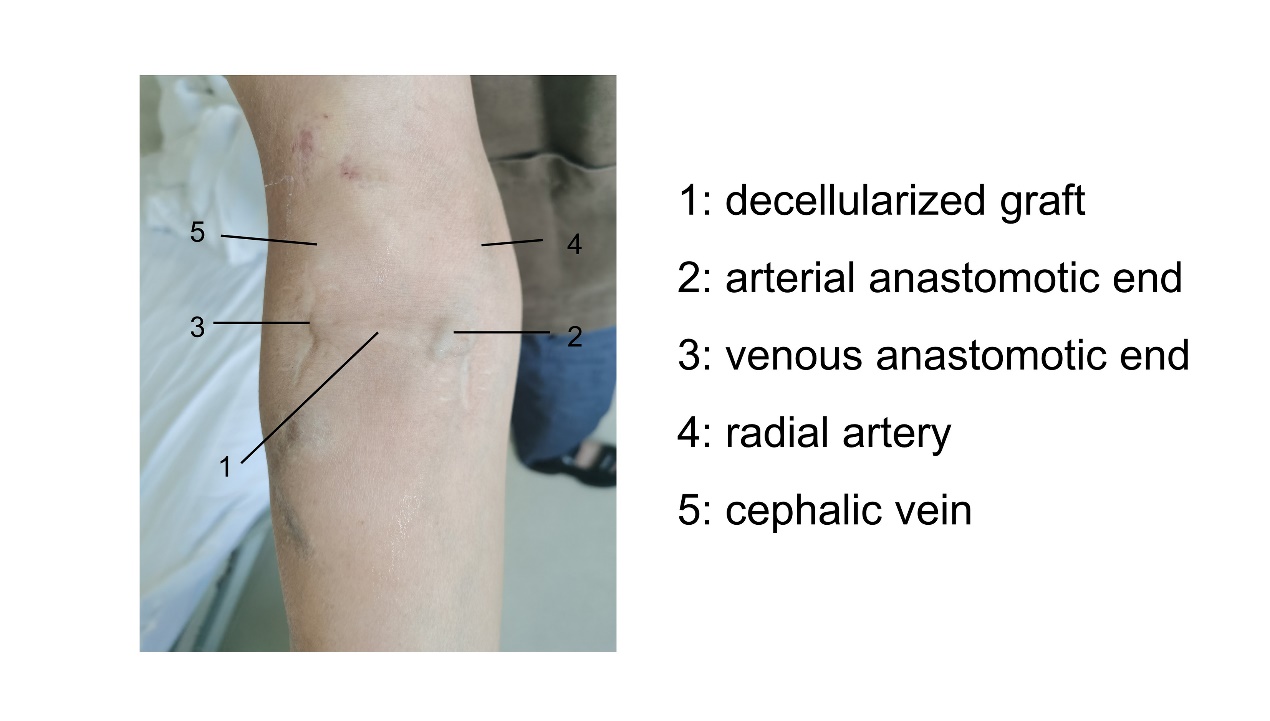


Supplementary figure 1 Evaluation of allogeneic decellularized graft in the human body five years post-implantation.


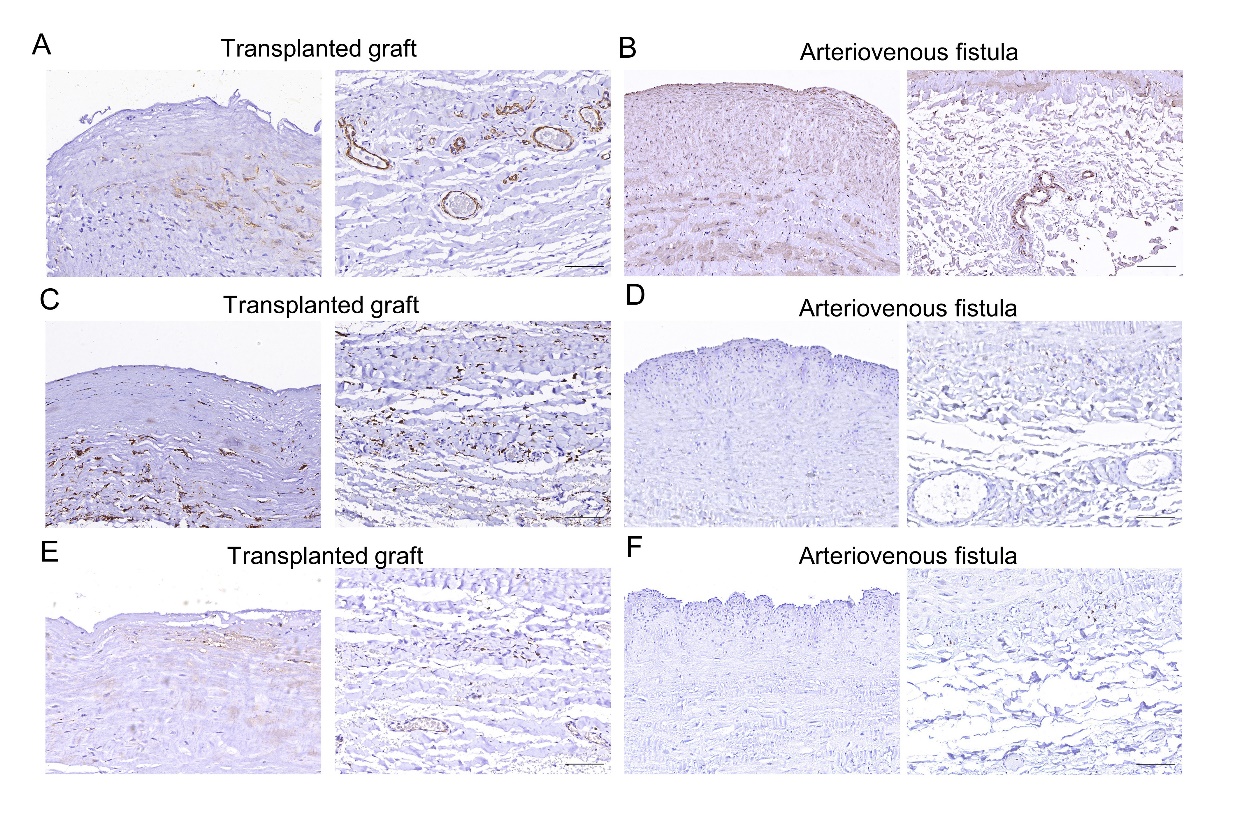


Supplementary figure 2 Immunohistochemistry (IHC) of α-SMA, CD68 and CD3. (A) IHC was used to assess the expression of α-SMA in the intima-media (left) and adventitia (right) of the transplanted graft (scale bar, 100μm). (B) IHC was used to assess the expression of α-SMA in the intima-media (left) and adventitia (right) of the AVF (scale bar, 100μm). (C) IHC was used to assess the expression of CD68 in the intima-media (left) and adventitia (right) of the transplanted graft (scale bar, 100μm). (D) IHC was used to assess the expression of CD68 in the intima-media (left) and adventitia (right) of the AVF (scale bar, 100μm). (E) IHC was used to assess the expression of CD3 in the intima-media (left) and adventitia (right) of the transplanted graft (scale bar, 100μm). (F) IHC was used to assess the expression of CD3 in the intima-media (left) and adventitia (right) of the AVF (scale bar, 100μm).


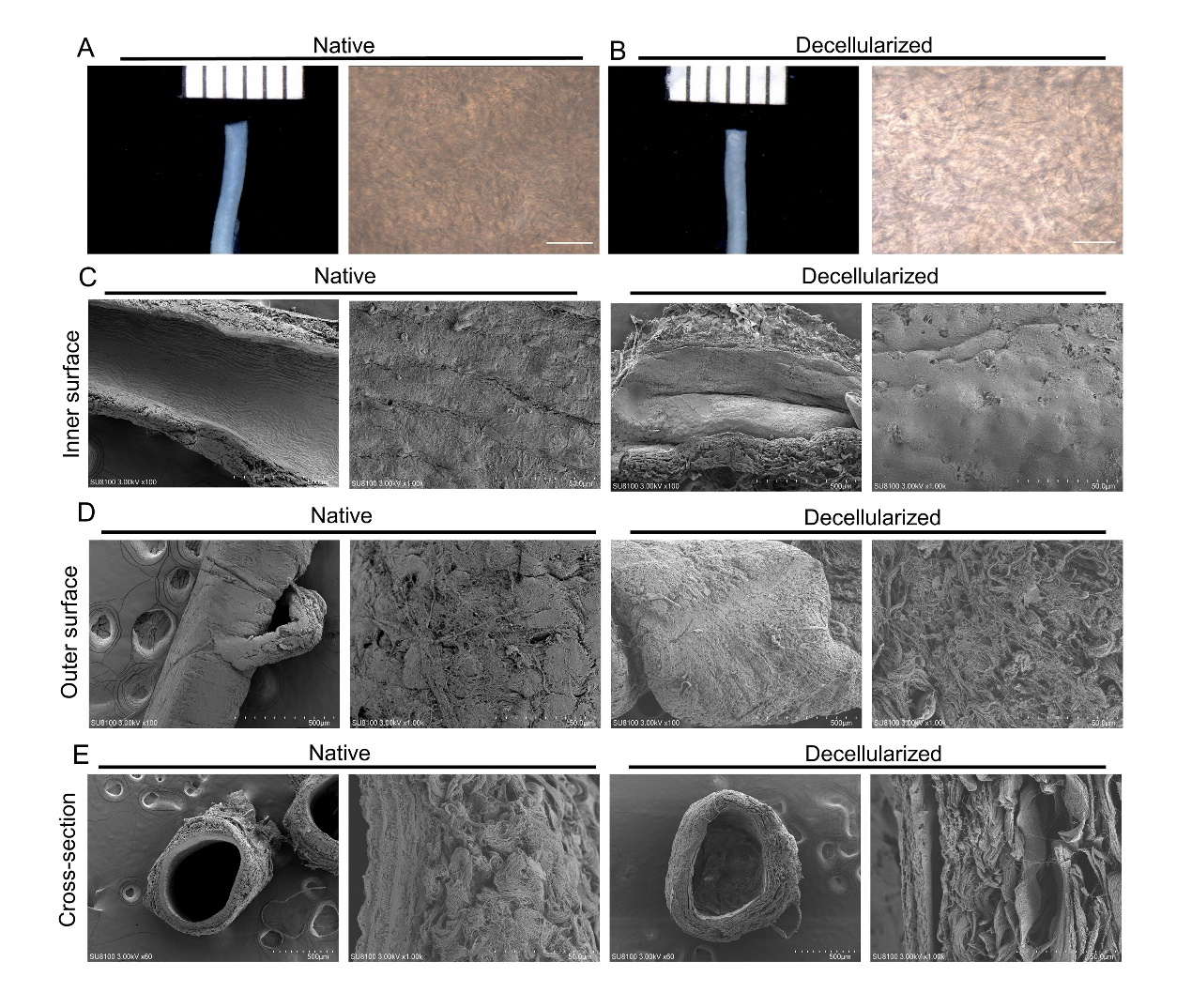


Supplementary figure 3 Gross morphology and scanning electron microscopy (SEM) of rat carotid artery before and after decellularization. (A-B) Gross morphology of native rat carotid artery (A) and decellularized rat carotid artery (B) (scale bar, 50μm). (C-E) SEM evaluations of the (C) internal surface (scale bar, 500μm and 50μm), (D) Outer surface (scale bar, 500μm and 50μm), and (E) cross-section (scale bar, 500μm and 50μm) of native rat carotid artery and decellularized rat carotid artery.


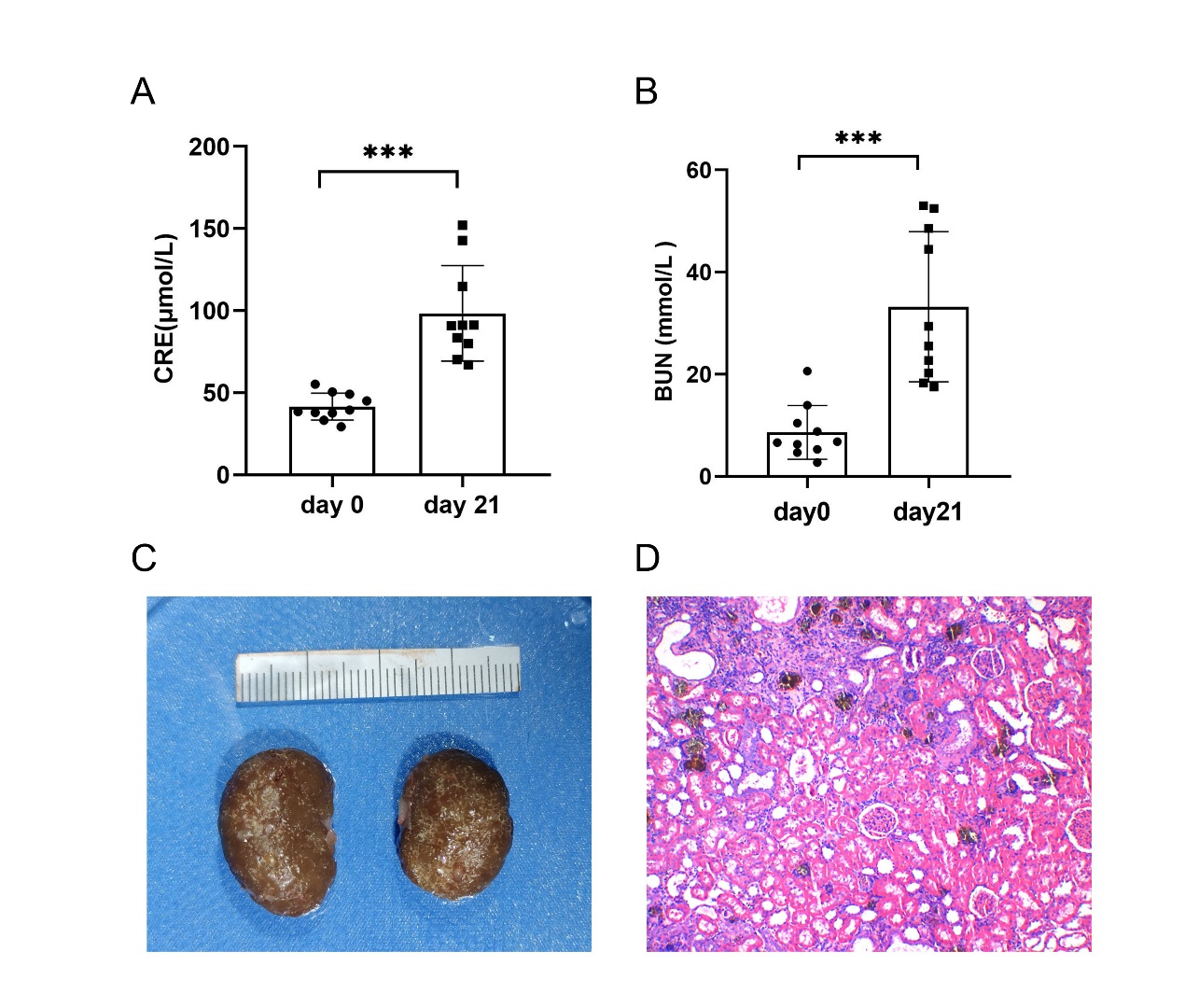


Supplementary figure 4 Results of rat renal failure model construction. (A) Changes in serum creatinine (CRE) levels before and after the construction of the rat renal failure model. (B) Changes in serum blood urea nitrogen (BUN) levels before and after the construction of the rat renal failure model. (C) Macroscopic view of rat kidneys in the renal failure model. Kidneys exhibit shrinkage with granular elevations on the surface. (D) HE staining of rat kidney sections from the renal failure model reveals interstitial fibrosis, tubular dilation and atrophy, as well as abnormal glomerular structures.


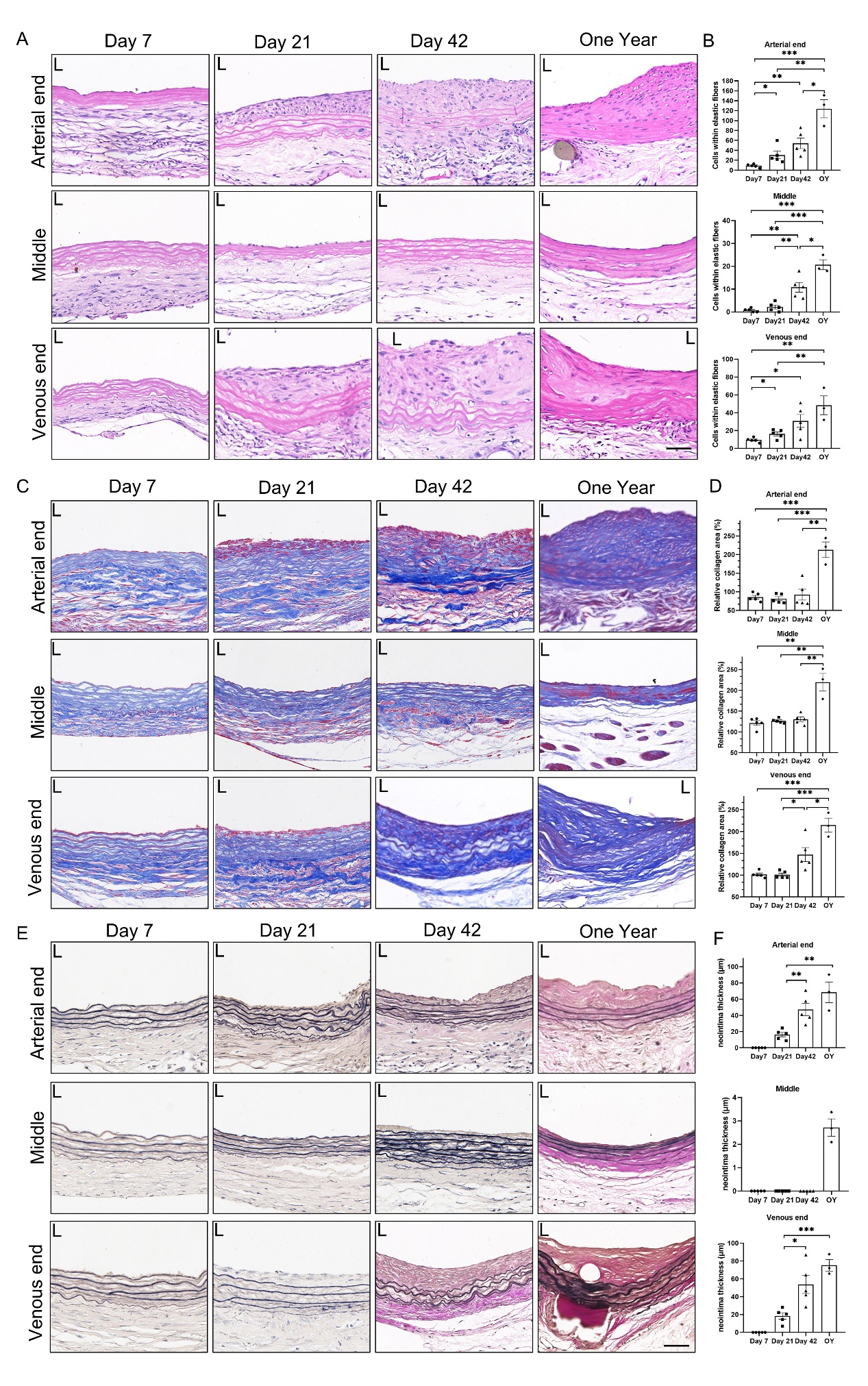


Supplementary figure 5 Haematoxylin and eosin (HE) staining, Masson's trichrome staining, and Verhoeff's Van Gieson (EVG) staining of postoperative decellularized vascular grafts. (A) Haematoxylin and eosin staining of decellularized vascular grafts at day 7, day 21, day 42 and one year (scale bar, 50 μm). (B) Quantification of the number of cells within the elastic fibers of arterial end (P<0.0001, ANOVA; day 7, day 21, day 42, n=5; one year, n=3), middle segment (P<0.0001, ANOVA; day 7, day 21, day 42, n=5; one year, n=3), and venous end (P=0.0017, ANOVA; day 7, day 21, day 42, n=5; one year, n=3). (C) Masson's trichrome staining of decellularized vascular grafts at day 7, day 21, and day 42; scale bar, 50 μm. (D) Quantification of collagen fiber content of arterial end (P<0.0001, ANOVA; day 7, day 21, day 42, n=5; one year, n=3), middle segment (P<0.0001, ANOVA; day 7, day 21, day 42, n=5; one year, n=3), and venous end (P<0.0001, ANOVA; day 7, day 21, day 42, n=5; one year, n=3). Because the region of the vascular adventitia lacks precise boundaries, only the intimal and medial layers of the blood vessel are taken into account. (E) Verhoeff's Van Gieson staining of decellularized vascular grafts at day 7, day 21, and day 42; scale bar, 50 μm. (F) Quantification of neointimal thickness of arterial end (P<0.0001, ANOVA; day 7, day 21, day 42, n=5; one year, n=3), middle segment (P<0.0001, ANOVA; day 7, day 21, day 42, n=5; one year, n=3), and venous end (P<0.0001, ANOVA; day 7, day 21, day 42, n=5; one year, n=3).


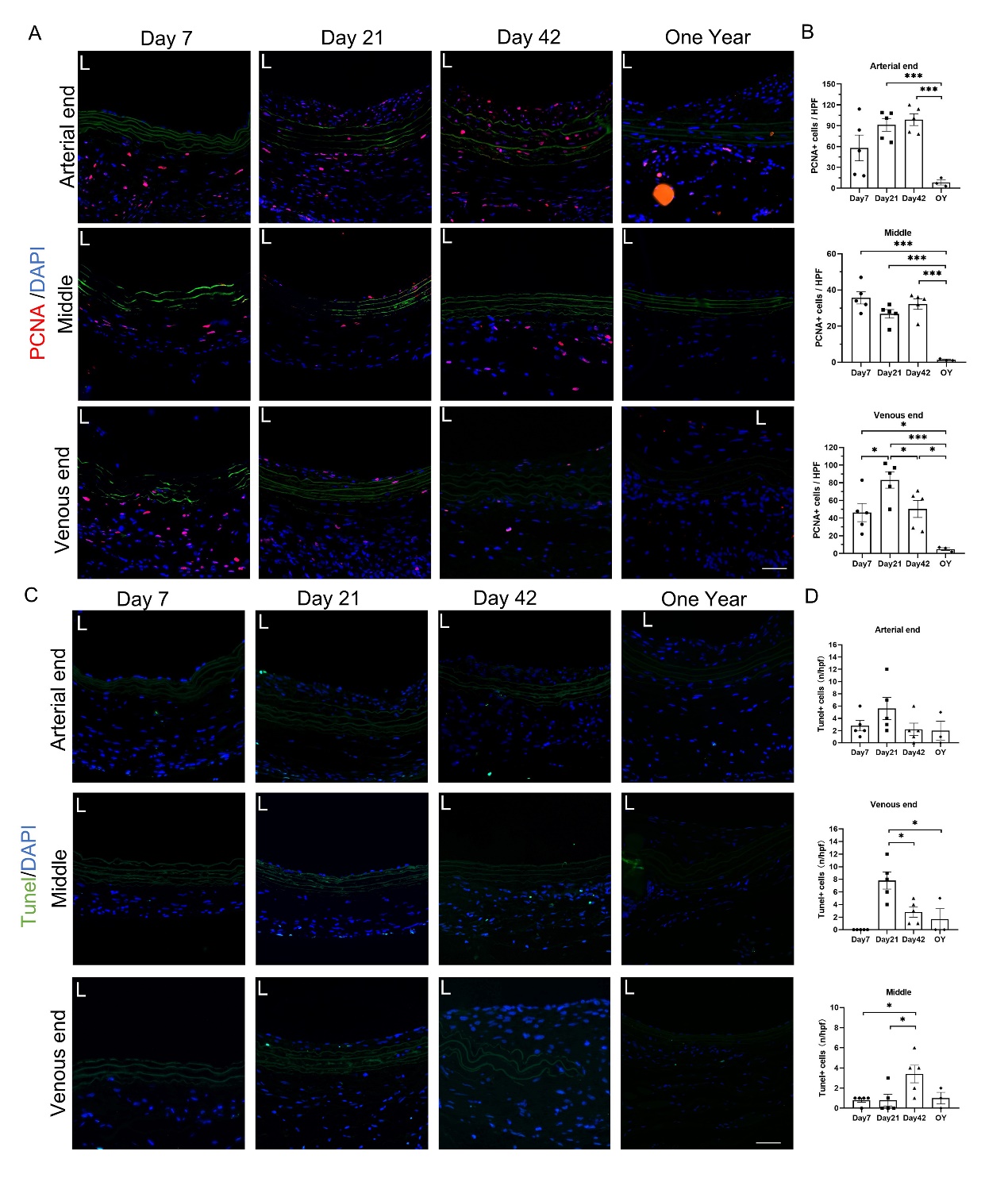


Supplementary figure 6 Evaluation of the proliferation and apoptosis status of cells in the decellularized vascular grafts and surrounding tissues. (A) Immunofluorescence staining of PCNA in the postoperative day 7, day 21, day 42, and one year; red indicates positive cells, and the green color represents the autofluorescence of elastic fibers (scale bar, 50 μm). (B) Statistical analysis of PCNA-positive cells of arterial end (P=0.0018, ANOVA; day 7, day 21, day 42, n=5; one year, n=3), middle segment (P<0.0001, ANOVA; day 7, day 21, day 42, n=5; one year, n=3), and venous end (P=0.0012, ANOVA; day 7, day 21, day 42, n=5; one year, n=3). (C) PCNA-positive cells at the site of elastic fiber fractures (scale bar, 50 μm). (D) Tunel staining in the postoperative day 7, day 21, day 42, and one year; green indicates positive cells (scale bar, 50 μm). (E) Statistical analysis of Tunel-positive cells of arterial end (P=0.2391, ANOVA; day 7, day 21, day 42, n=5; one year, n=3), middle segment (P=0.0215, ANOVA; day 7, day 21, day 42, n=5; one year, n=3), and venous end (P=0.0004, ANOVA; day 7, day 21, day 42, n=5; one year, n=3).


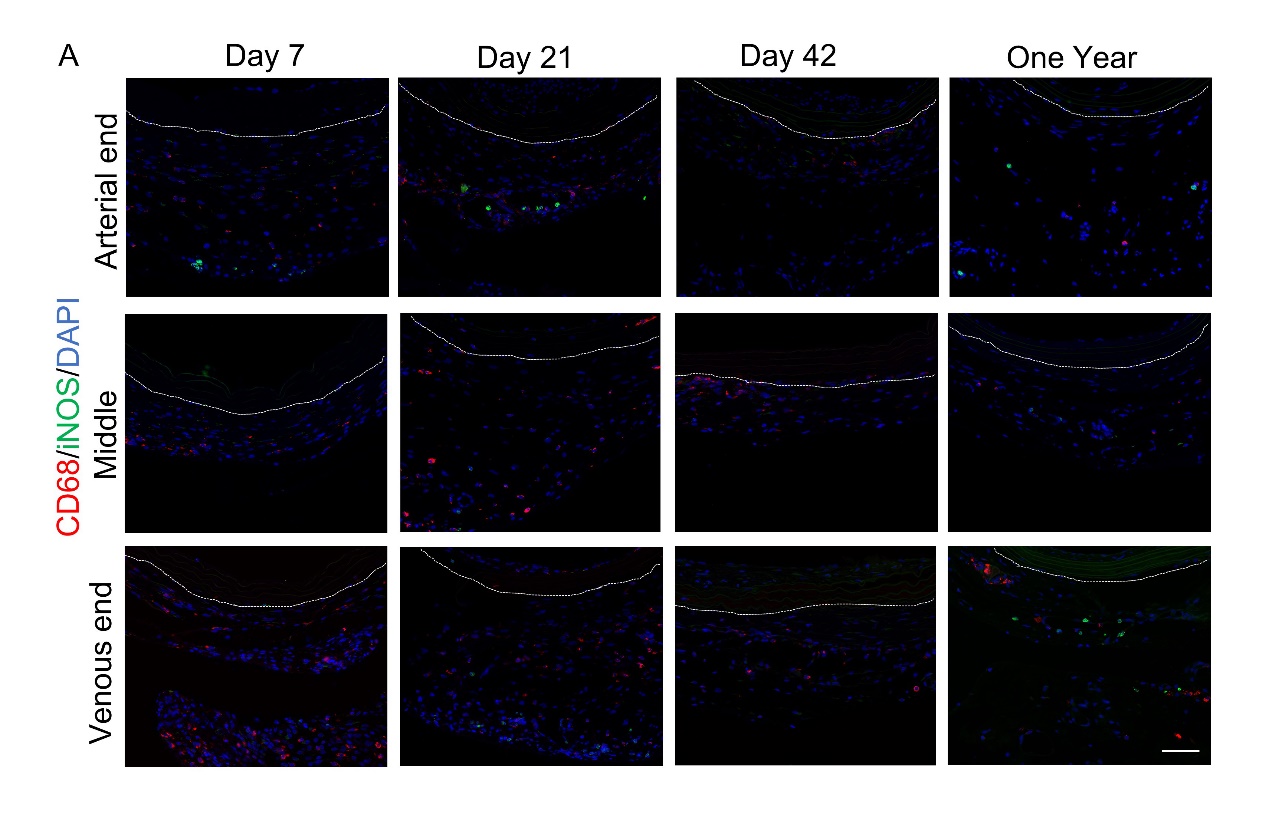


Supplementary figure 7 Accumulation of M1 macrophages around the decellular vascular graft. (A) M1 macrophages around the decellular vascular graft (scale bar, 50 μm). The area outside the dashed lines represents the outer of decellularized vascular grafts.
